# ACLY and ACC1 Regulate Hypoxia-Induced Apoptosis by Modulating ETV4 via α-ketoglutarate

**DOI:** 10.1371/journal.pgen.1005599

**Published:** 2015-10-09

**Authors:** Melissa M. Keenan, Beiyu Liu, Xiaohu Tang, Jianli Wu, Derek Cyr, Robert D. Stevens, Olga Ilkayeva, Zhiqing Huang, Laura A. Tollini, Susan K. Murphy, Joseph Lucas, Deborah M. Muoio, So Young Kim, Jen-Tsan Chi

**Affiliations:** 1 Department of Molecular Genetics and Microbiology, Duke University Medical Center, Durham, North Carolina, United States of America; 2 Center for Genomic and Computational Biology, Duke University Medical Center, Durham, North Carolina, United States of America; 3 Department of Electrical and Computer Engineering, Duke University Medical Center, Durham, North Carolina, United States of America; 4 Sarah W Stedman Nutrition and Metabolism Center, Duke University Medical Center, Durham, North Carolina, United States of America; 5 Duke Institute of Molecular Physiology, Duke University Medical Center, Durham, North Carolina, United States of America; 6 Department of Obstetrics and Gynecology, Division of Gynecologic Oncology, Duke University Medical Center, Durham, North Carolina, United States of America; McMaster University, CANADA

## Abstract

In order to propagate a solid tumor, cancer cells must adapt to and survive under various tumor microenvironment (TME) stresses, such as hypoxia or lactic acidosis. To systematically identify genes that modulate cancer cell survival under stresses, we performed genome-wide shRNA screens under hypoxia or lactic acidosis. We discovered that genetic depletion of acetyl-CoA carboxylase (*ACACA* or *ACC1*) or ATP citrate lyase (*ACLY*) protected cancer cells from hypoxia-induced apoptosis. Additionally, the loss of ACLY or ACC1 reduced levels and activities of the oncogenic transcription factor ETV4. Silencing ETV4 also protected cells from hypoxia-induced apoptosis and led to remarkably similar transcriptional responses as with silenced ACLY or ACC1, including an anti-apoptotic program. Metabolomic analysis found that while α-ketoglutarate levels decrease under hypoxia in control cells, α-ketoglutarate is paradoxically increased under hypoxia when ACC1 or ACLY are depleted. Supplementation with α-ketoglutarate rescued the hypoxia-induced apoptosis and recapitulated the decreased expression and activity of ETV4, likely via an epigenetic mechanism. Therefore, ACC1 and ACLY regulate the levels of ETV4 under hypoxia via increased α-ketoglutarate. These results reveal that the ACC1/ACLY-α-ketoglutarate-ETV4 axis is a novel means by which metabolic states regulate transcriptional output for life vs. death decisions under hypoxia. Since many lipogenic inhibitors are under investigation as cancer therapeutics, our findings suggest that the use of these inhibitors will need to be carefully considered with respect to oncogenic drivers, tumor hypoxia, progression and dormancy. More broadly, our screen provides a framework for studying additional tumor cell stress-adaption mechanisms in the future.

## Author Summary

During the development of most solid tumors, there are characteristic physiological differences in the tumor that result from tumor cells outgrowing their local blood supply. Two of these physiological differences, or “stresses,” that occur in the tumor are low oxygen levels (hypoxia) and an accumulation of lactic acidic (lactic acidosis). Cancer cells experiencing hypoxia and lactic acidosis tend to be more resistant to chemo- and radio-therapy and metastasize more readily. Therefore, it is important to understand how tumor cells adapt to and survive these stresses. We used a large scale screening experiment in order to find which genes and proteins are involved in tumor cell adaptation and survival under hypoxia or lactic acidosis. We found that inhibiting either of two genes involved in lipid synthesis allowed tumor cells to survive hypoxia. This occurred because silencing these genes led to an increase in the metabolite α-ketoglutarate, which repressed a transcription factor that contributed to cell death under hypoxia. This research specifically advances our understanding of how tumor cells survive hypoxia and lactic acidosis and more broadly enhances our understanding of the cellular biology of solid tumors.

## Introduction

Most solid tumors have substantial physiological deviations from normal tissue, which manifest as tumor microenvironment (TME) stresses [1,2]. These TME “stresses” include, among others, the limited availability of oxygen (hypoxia), glucose or amino acids, and an accumulation of lactic acid (lactic acidosis). In order to grow and propagate a solid tumor, tumor cells must adapt to and survive under these TME stresses. Additionally, tumor cells in regions of LA or hypoxia are more radio- and chemo-resistant and are more likely to metastasize [3]. Since TME stresses are found in most solid tumors, targeting stress-adaptation mechanisms of tumor cells may offer a significant therapeutic window to selectively eradicate tumor cells and improve patient outcomes [4]. Yet, current therapies targeting cells specifically under stress have significant limitations. For example, angiogenesis is a well-established, valuable therapeutic target with agents developed to block it at various stages of tumor development. However, many anti-angiogenic therapies fail over time, through acquired or inherited resistance that may involve the presence of tumor hypoxia [5]. Therefore, a number of other strategies are being developed to directly target hypoxic cells, such as blocking lactate transporters [6,7], or pro-drugs that are activated only in the presence of low oxygen [4]. There remains significant room for improvement to target cells under stress, and thus there remains a need to better understand the genes that impact cellular survival under TME stresses.

Tumor cells employ at least two kinds of adaptive strategies to cope with TME stresses, transcriptional and metabolic, and these are often interconnected. Transcriptional changes are mediated by stress-activated transcription factors. For example, the most significant factors known to regulate a cell’s hypoxia response are the hypoxia-inducible factors (HIFs), which are stabilized under low oxygen and mediate complex transcriptional programs that increase glucose uptake and enhance glycolysis [8,9]. HIF–1α also regulates glutamine metabolism by affecting the ubiquitination of its oxidizing enzyme, AKGDH, to promote reductive carboxylation of glutamine under hypoxic conditions [10–12]. In addition to the HIFs, many other transcription factors regulate cellular responses to stresses in the TME. For example, the MondoA:Mlx complex senses and initiates transcriptional changes under both glucose deprivation and lactic acidosis to induce TXNIP and restrict glucose uptake [13–15]. Importantly, multiple co-factors and modulators add to the complexity of stress mediated transcriptional responses [16]. While these adaptive mechanisms can be successful to sustain cell growth under stress, in multiple contexts hypoxia induces apoptosis [17–19].

Transcriptional responses mediate many metabolic reprogramming events, but, recently, it is also becoming evident that metabolic events can regulate gene expression. For example, the metabolic enzyme ATP citrate lyase (ACLY) generates glucose-derived acetyl-CoA from citrate to alter histone acetylation and, therefore, transcriptional activation [20]. Importantly, the metabolically sensitive mTOR signaling cascade can activate HIF, even under normoxia [21,22]. Mutations in succinate dehydrogenase and fumarate hydratase both lead to increased levels of their substrates (succinate and fumarate, respectively), causing increased HIF–1α stability and alterations of genome-wide histone and DNA methylation [23,24]. Metabolites such as NAD+, NOS and α-ketoglutarate (α-KG) can also affect HIF function and histone and DNA modifications [21,22,25–28]. While cellular metabolism and transcriptional changes can provide flexibility for adaptation, cancer cells can also become reliant on, and thus vulnerable to the inhibition of, specific metabolic pathways or gene products [29–31]. Therefore, a better understanding of the genes necessary for modulating cancer cell survival under TME stresses will improve the development of targeted therapies that selectively eradicate cancer cells under stresses [4,32].

Functional genetic screens provide an unbiased and powerful means of identifying genes responsible for any phenotype that can be measured experimentally. Unbiased RNAi screens have identified genes that influence the survival of organisms and cells under various stresses [33–36]. These studies provide a foundation for using RNAi screens to uncover genes involved in TME-relevant stress responses, but so far have not been applied genome-wide to identify genes that modulate the survival of cancer cells under hypoxia or lactic acidosis. To better understand the genes involved in the adaptations of cancer cells under TME stresses, we performed genome-wide pooled shRNA screens of lung cancer cells under hypoxia and lactic acidosis. Completing these screens revealed that the inhibition of ACC1 or ACLY, two key enzymes of *de novo* lipogenesis, protected cancer cells from hypoxia-induced apoptosis. ACC1 or ACLY inhibition protected cells by elevating levels of α-ketoglutarate under hypoxia to reduce the activity of the oncogenic transcription factor ETV4. Together, these data provide evidence to support a molecular connection between cellular metabolic and transcriptional hypoxia adaptation via the ACLY-ACC1-ETV4 axis through α-ketoglutarate.

## Results

### Genome-wide pooled shRNA screens in hypoxia and lactic acidosis

**Fig 1:**
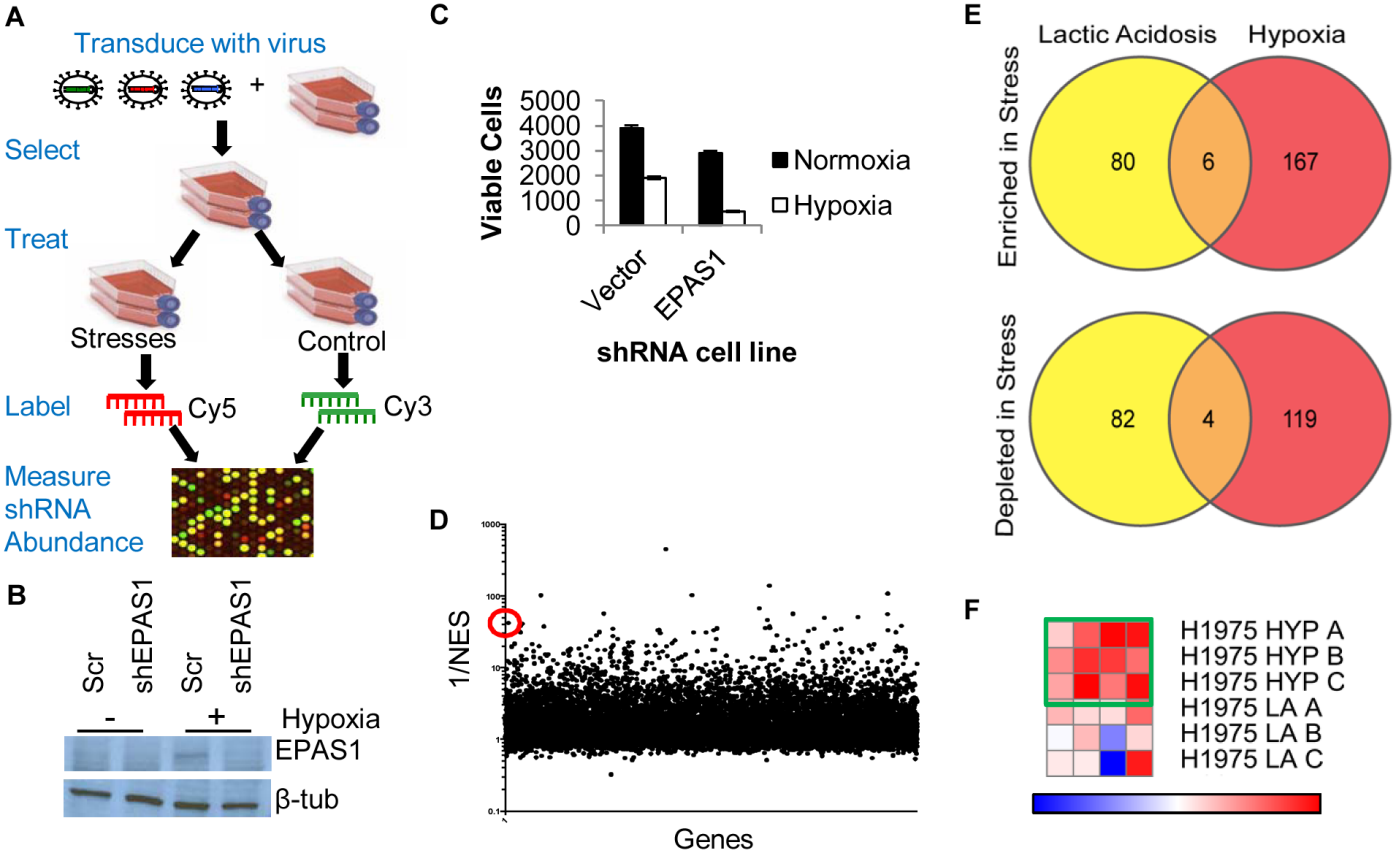
Genome-wide shRNA screen identifies the depletion of ACC1 as protective for cells under hypoxia. (A) The overall design of genome-wide pooled shRNA screen protocol. Each treatment was performed in n = 3. (B) Western blot showing decreased protein level expression of EPAS1 (HIF–2α) by shRNA. (C) Viable cell number counts by trypan blue exclusion after indicated cells in 4 days of hypoxia (n = 9). (D) Analysis of screen data by RIGER with GENE-E program with the probability of a gene being a hit indicated by 1/NES (normalized enrichment score). With the second best hairpin method, ACC1 is 13^th^ best performing gene overall (indicated by red circle). (E) Venn diagrams showing the overlap of genes in the multiple hairpin analysis (see Methods) across both TME stress treatments, separated by shRNA enrichment (top) or depletion (bottom) in the stress condition. (F) GENE-E heat map results of ACC1 hairpins in hypoxia screen (top 3 rows) and lactic acidosis screen (bottom 3 rows). Each row is one biological replicate (n = 3 per treatment). Each column is a different shRNA targeting ACC1. Green box highlights the hypoxia result where the gene is predicted as a “protective” hit.

To identify genes that modulate cell survival under lactic acidosis and hypoxia, we conducted genome-wide, shRNA-based, contextual pooled screens in the lung cancer cell line H1975 under hypoxia or lactic acidosis (Fig 1a). To preferentially discover genes important for survival rather than proliferation, the screen was done in low proliferative conditions (see Methods). Cells were transduced with a genome-wide MSCV-based shRNA library [37], selected for successful transduction with puromycin and then grown under control (21% O_2_, pH 7.4), hypoxia (2% O_2_, pH 7.4), or lactic acidosis (21% O_2_, 25mM lactic acid, pH 6.7) for 4 days, as performed previously [15,38]. At these stress treatments, there was a ~50% reduction in cell number, which allowed us to uncover both genes whose suppression reduced or improved survival under stresses. Genomic DNA was isolated from cells under each condition and the genome-incorporated shRNA sequences were amplified by PCR. The amplified PCR products were labeled and competitively hybridized to a custom microarray to identify those shRNA sequences that were either enriched or depleted relative to the control treatment (Fig 1a). The custom array was modified slightly from similar arrays used in other shRNA screens [39,40]. When different ratios of differentially labeled PCR products were hybridized on the arrays, we noted distinguishable differences in the signals, demonstrating the specificity and sensitivity of the array (S1a Fig). Biological triplicates of each condition had highly reproducible signals (S1b Fig). The abundance of each shRNA sequence reflected the effect of its target gene on cell survival under stresses: if the shRNA was depleted in the stress treatment, the gene it targeted had a “synthetic sick/lethal” phenotype; if the shRNA was enriched in the stress treatment, the gene it targeted had a “synthetic survival/protective” phenotype under stress. In order to analyze the effect of each shRNA in stress, we calculated an “R/G” ratio (see Methods). R/G ratios were distributed on a scale of +/- 4.0 that was highly consistent between replicates and stresses (S1c Fig).

To minimize false positives due to off-target effects of individual shRNAs, we focused only on the genes that had at least two distinct shRNA sequences that were enriched or depleted (S1 and S2 Tables, see Methods). Importantly, this “multiple hairpin analysis” identified *EPAS1* (hypoxia-inducible factor 2α, HIF–2α) as a synthetic lethal gene under hypoxia. We further validated this by showing that silencing EPAS1 by shRNA reduced cell survival under hypoxia (Fig 1b and 1c). This result was consistent with the critical role of EPAS1 in cellular adaptation to hypoxia [8]. The “re-discovery” of *EPAS1* provided confidence in our screen and analysis methods. However, no pathways or gene ontology groups were significantly enriched within the different categories of “multiple hairpin hits”. We then performed a RIGER analysis using a log-fold change and the second best shRNA for each gene criteria (Fig 1d, S3 Table) [41]. This RIGER analysis revealed an enrichment for genes affecting mRNA regulation and binding, as well as membrane dynamics and nuclear localization [42]. Additionally, there was little overlap between the genes targeted by multiple hairpins, either enriched or depleted, in the two stresses (Fig 1e). This was consistent with past reports of distinct responses and adaptations to hypoxia and lactic acidosis [15,43].

### Genome-wide screen identified ACC1 depletion as protective to cells under a hypoxic stress

Next, we identified high confidence “hits” in both the multiple hairpin and RIGER analyses for further investigation. From these considerations, we chose *ACC1* (acetyl-CoA carboxylase 1 or *ACACA*) as the top candidate. *ACC1* encodes the cytosolic isoform of acetyl-CoA carboxylase, which converts acetyl-CoA to malonyl-CoA in the rate-limiting step of *de novo* fatty acid synthesis. There was an enrichment of shRNAs targeting ACC1 in the hypoxic versus the control condition, suggesting that ACC1 knockdown allowed for improved survival under hypoxia. *ACC1* had 4 hairpins enriched under hypoxia (Fig 1f) and scored as the 13^th^ best gene in the RIGER analysis using the second best shRNA metric (Fig 1d). Additionally, the down-regulation of *ACC1* was previously shown to protect cancer cells from glucose deprivation and matrix detachment stresses [44]. Together, these data prompted us to validate and investigate the role of ACC1 under hypoxia.

**Fig 2:**
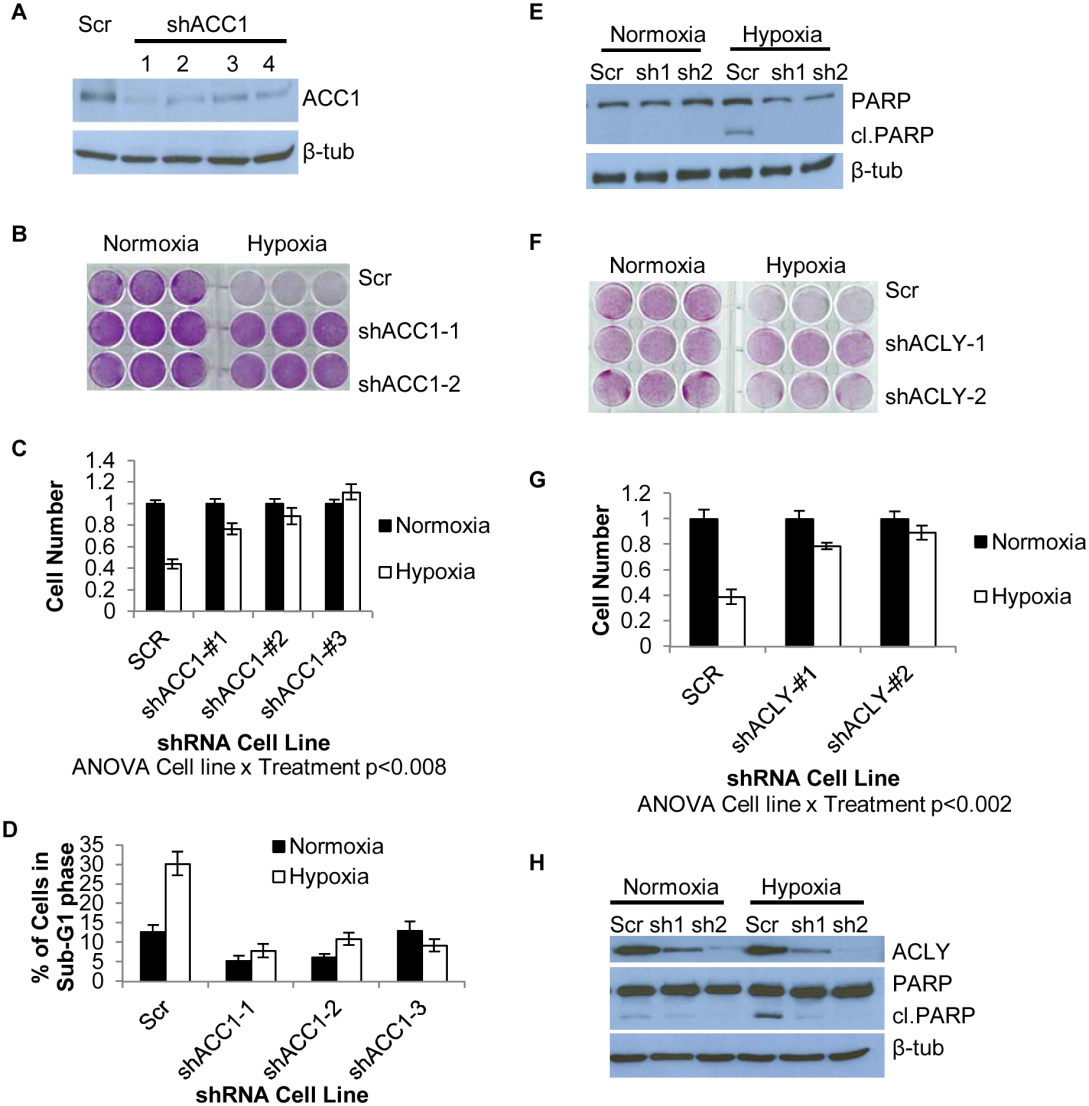
Depletion of ACC1 or ACLY protects cells from hypoxia-induced apoptosis. (A) Western blot showing efficiency of 4 independent shRNAs targeting ACC1 in H1975 cells. (B) Crystal violet staining of scramble and ACC1 shRNA cells after 4 days of hypoxia. (C) Viable cell numbers of indicated cells after 4 days of hypoxia as determined by counting nuclei (n = 9). (D) Percent of cells in the sub-G1 phase as determined by PI staining and FACS analysis of indicated cell line after 4 days of hypoxia (n = 9). (E) Western blot showing cleaved PARP levels with scramble and shACC1 cells after hypoxia for 48 hours. (F) Crystal violet staining of scramble and shACLY cell lines after 4 days of hypoxia. (G) Viable cell number determined by trypan blue exclusion after 4 days of hypoxia (n = 3). (H) Western blot showing ACLY protein levels and PARP cleavage in the indicated cells after 48 hours of hypoxia. Data are represented as mean values +/- SEM. Data are from the H1975 cell line.

To validate the shRNA screen result, we silenced ACC1 expression through lentiviral infection of multiple shRNAs that targeted different sequences from those shRNAs used in the screen (see Methods). We confirmed the successful reduction of ACC1 protein by these shRNAs (Fig 2a). In the control cells transduced with a scramble shRNA, hypoxia significantly decreased cell viability and induced apoptosis (Fig 2). However, silencing ACC1 by multiple shRNAs inhibited the hypoxia-induced apoptosis as shown by crystal violet staining (Fig 2b), cell counting (Fig 2c), propidium-iodide staining (flow cytometry) (Fig 2d) and PARP cleavage (Fig 2e). This hypoxic protection associated with ACC1 silencing was also reproduced in additional hypoxia-sensitive cell lines, including MDA-MB–231 (breast cancer; S2a and S2b Fig), and PANC–1 (pancreatic cancer; S2e and S2g Fig). Furthermore, chemical inhibition of ACC1 through the AMPK agonist metformin also protected H1975 cells from hypoxia-induced apoptosis (S2h and S2i Fig). Collectively, these data successfully validated the screen results and showed that the depletion of ACC1 enhanced cell survival under hypoxia in multiple cancer cells from different tissues of origin.

### Inhibition of ACC1 or ACLY inhibits hypoxia-induced apoptosis

We next investigated the specificity of the hypoxia protection by ACC1 depletion. Besides *ACACA* (*ACC1*), *ACACB* (*ACC2*) encodes another isoform of acetyl-CoA carboxylase, located in the outer mitochondrial membrane [45]. *ACC2* was not a hit in our screen and its silencing by shRNA did not offer a similar hypoxia protection as seen with ACC1 depletion (S2j Fig). Next, we determined whether depletion of ACC1 protected against other TME stresses (lactic acidosis, glutamine deprivation and glucose deprivation). We found that the protective effect of shACC1 was seen only under hypoxia (S2k and S2l Fig). Since the HIFs are the major transcriptional responders to hypoxia, we examined how loss of ACC1 affected HIF–1α levels. Interestingly, we found decreased levels of HIF–1α under hypoxia with ACC1 depletion across multiple cell lines (S3a–S3c Fig). These data suggested that the protective phenotype was not due to upregulation of the HIF response. Overall, these results indicated that only the cytosolic isoform of acetyl-CoA carboxylase (encoded by *ACC1*) was essential for apoptosis, specifically under a hypoxic stress.

When we examined the effect of blocking enzymes up- or downstream of ACC1, we found that silencing ATP citrate lyase (ACLY) also enhanced survival under hypoxia (Fig 2f and 2g). *ACLY* encodes the enzyme immediately upstream of ACC1 in lipogenesis, catalyzing the formation of acetyl-CoA and oxaloacetate from citrate. Similar to ACC1, this protection results from the inhibition of hypoxia-induced apoptosis (Fig 2h). The protective effect of ACLY depletion was also reproduced in MDA-MB–231 cells (S2c and S2d Fig) and PANC–1 cells (S2f and S2g Fig). In H1975 and MDA-MB–231 shACLY cells there was also decreased HIF–1α expression under hypoxia as was seen in the shACC1 cells (S3d and S3e Fig). These data showed that blocking lipogenesis at the points of either ACLY or ACC1 inhibited apoptosis and permitted cell survival under hypoxia in cells of multiple tissue types.

### Loss of ACC1 or ACLY did not protect cells from hypoxia-induced apoptosis through NADPH conservation to relieve oxidative stress

Lipogenesis is a highly anabolic process that requires significant amounts of NADPH and ATP. Previously, silencing ACC1 protected cells from death caused by glucose deprivation and matrix detachment by preserving NADPH and ATP to counteract the ensuing oxidative stresses [44,46]. We tested the relevance of these factors in our system. In H1975 cells, silencing ACC1 trended toward increasing the NADP+/NADPH ratio, suggesting a decrease in available NADPH (S4a Fig). We reasoned that if the NADPH were being used to combat elevated reactive oxygen species under hypoxia, then supplementation with antioxidants should protect control cells from hypoxia-induced death similar to [44]. However, neither the addition of N-acetyl cysteine nor glutathione antioxidants rescued hypoxia-induced death in control cells (S4b and S4c Fig). While ATP levels were higher with ACC1 silenced, the change in ATP levels from normoxia to hypoxia was consistent in control and knockdown cells and thus could not readily explain the hypoxia protection (S4d Fig). Therefore, in these cells with ACC1 or ACLY depleted, changes in NADPH and ATP levels may not be the primary mechanism for cell survival under hypoxia. Therefore, we sought to identify another mechanistic explanation for this hypoxia protection phenotype.

### Depletion of the transcription factor ETV4 led to similar hypoxia-protection and gene expression phenotypes as with the loss of ACC1 or ACLY

**Fig 3:**
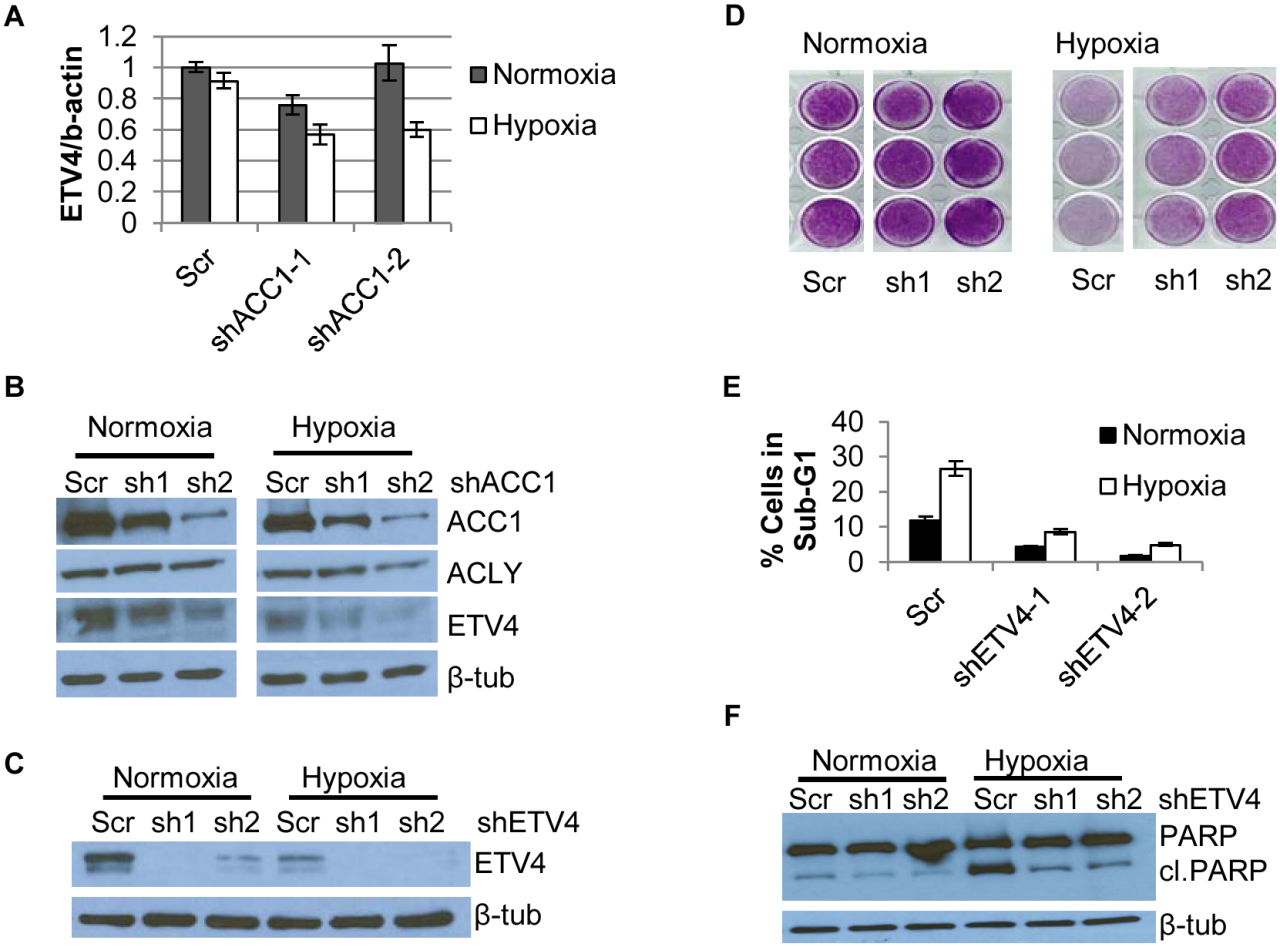
Depletion of ETV4 protects cells from hypoxia-induced apoptosis. (A) qPCR analysis of ETV4 mRNA levels in shACC1 cells in normoxia and hypoxia (n = 6). (B) Western blot of ETV4 protein levels in shACC1 cell lines under normoxia and hypoxia. (C) Western blot of ETV4 protein levels in shETV4 cell lines under normoxia and hypoxia. (D) Crystal violet staining of two distinct shETV4 cell lines after 4 days of hypoxia. (E) Percent of cells in the sub-G1 phase as determined by PI staining of indicated cell line after 4 days of hypoxia (n = 6). (F) Western blot of intact and cleaved PARP in shETV4 cell lines under normoxia or hypoxia. Data are represented as mean values +/- SEM. Data are from the H1975 cell line.

In our “multiple hairpin analysis” of the hypoxia genome-wide screen, there was an enrichment of shRNAs targeting a PEA3 transcription factor family member, *ETV4*. These results suggested that silencing ETV4 may be protective under hypoxia. A link between lipogenesis and ETV4 was previously established when levels of malonyl-CoA were associated with ETV4 activity [47]. This prompted us to investigate a potential regulatory relationship between ACLY, ACC1 and ETV4. Real-time PCR analysis showed that hypoxia led to a reduction of ETV4 mRNA in the ACC1-depleted, but not control cells (Fig 3a). Additionally, we noted correspondingly reduced ETV4 protein in the shACC1 cells as compared to the scramble cells (Fig 3b). Reduced ETV4 mRNA and protein levels were also noted in the ACLY-depleted cells (S4e and S4f Fig). While ETV4 protein levels were somewhat decreased under normoxia, the down-regulation was stronger under hypoxia. This regulation was mostly specific to ETV4; the other PEA3 subfamily members, ETV1 and ETV5, were not consistently altered by ACC1 depletion (S4g Fig). Additionally, neither ETV1 nor ETV5 were identified as “multiple hairpin hits” in the shRNA screen. We validated the hypoxia-protective phenotype of ETV4 loss with two different shRNAs targeting ETV4 (Fig 3c and 3d). Similar to ACC1/ACLY depletion, the depletion of ETV4 also reduced the percentage of cells in the sub-G1 phase (Fig 3e) and decreased PARP cleavage (Fig 3f) under hypoxia. These data showed that the loss of ETV4 decreased hypoxia-induced apoptosis, similar to the phenotype of reduced ACC1 or ACLY.

**Fig 4:**
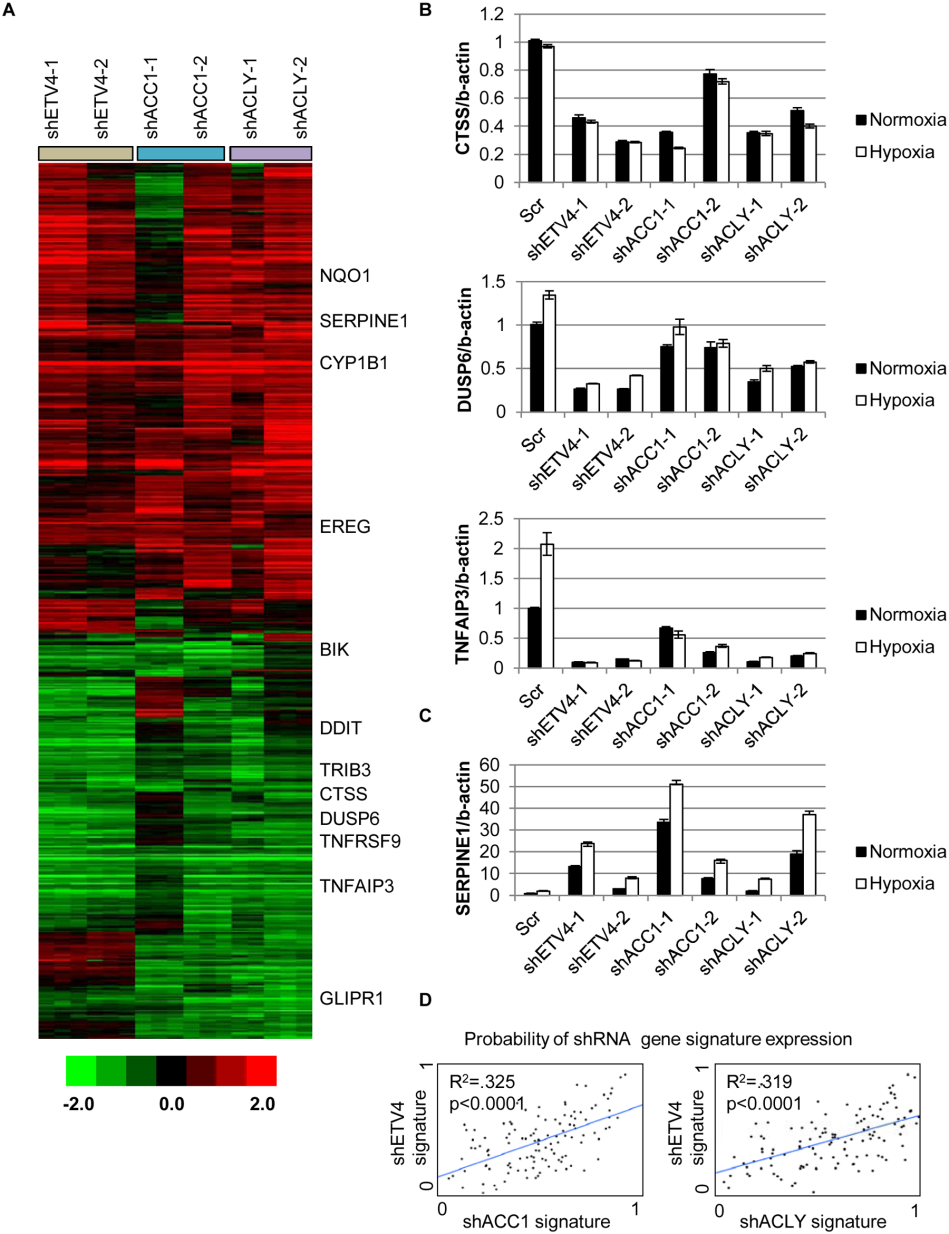
Silencing ETV4 triggers similar gene expression changes as loss of ACC1 or ACLY. (A) Heat map of transcriptional responses to the depletion of indicated genes (ETV4, ACC1 or ACLY) by two shRNAs each (n = 3 per shRNA) under hypoxia. Data are log2 values, zero transformed to the scramble shRNA cells. Filtering criteria of at least six occurrences with values greater than 1.7 resulted in 641 probesets. (B,C) qPCR validation of mRNA changes of similarly (B) down-regulated and (C) up-regulated genes with depletion of ETV4, ACLY or ACC1 (n = 6). (D) Binary regressions of shACC1 (left panel) or shACLY (right panel) and shETV4 gene signatures when compared across gene expression patterns of 130 breast cancer tumor samples (see text and Methods).

Since ETV4 is a transcription factor, we investigated the contribution of reduced ETV4 activity to the transcriptional response of *ACLY* or *ACC1* depletion. We used microarrays to analyze the global transcriptional response to the silencing of each ACC1, ACLY or ETV4 by two independent shRNAs under hypoxia (each shRNA was done in triplicate). The transcriptional responses were determined by zero-transformation against the shScramble cells [48]. Next, the data were filtered with a 1.7-fold change in at least six arrays and the selected 641 probesets were grouped by hierarchical clustering (Fig 4a). This analysis revealed a remarkable similarity between the transcriptional responses to the depletion of ETV4, ACC1 or ACLY with the induction and repression of common sets of genes (Fig 4a). Using the GATHER [49] algorithm, we noticed an “anti-apoptotic expression program” that included both the induction of negative regulators of apoptosis signaling such as NQO1, CYP1B1 and SERPINE1 [50,51] and the repression of the apoptosis-promoting genes BIK, TNFRSF9, TNFAIP3, GLIPR1, DDIT and TRIB3 (Fig 4a). The induction and repression of multiple genes in the shACC1, shACLY and shETV4 cells were confirmed by real-time qPCR (Fig 4b and 4c). Using GSEA, these gene expression changes were highly overlapping in all pairwise comparisons with both up- and down-regulated genes (S4h and S4i Fig).

Next, we evaluated whether the ACC1-affected genes were transcriptional targets of ETV4 by two different approaches. First, we compared publicly available ETV4 ChIP-seq data (Cistrome Finder [52]) from PC3 cells [53] with the genes that were changed in our microarray analysis of H1975 cells with loss of ETV4, ACC1 or ACLY (Fig 4a). While performed in a different cell (PC3), these analyses still identified at least two potential direct ETV4 target genes, PLEC (S5a Fig) and DUSP6 (S5b Fig). For both genes, there were peaks indicating direct ETV4 binding that overlapped with histone H3 lysine 27 acetylation (a mark of actively transcribed gene bodies) and DNase hypersensitive regions of open chromatin (S5a and S5b Fig). DUSP6 has been previously described as an ETS transcription factor family target [54,55]. While PLEC was reported to interact with vimentin, an ETV4 direct target [56], this analysis suggested that PLEC itself may represent a novel ETV4 target. In the second approach, we used qPCR to determine if the ACC1-affected genes could be “rescued” by ETV4 over-expression. We found that CTSS, COL13A1, DUSP6 and SERPINE1 could be reversed by ETV4 re-expression (S6a and S6b Fig). In contrast, the ACC1-altered expression of other genes was either partially or not restored upon ETV4 overexpression (S6c Fig). This analysis suggested that some of the gene expression changes discovered by our microarray analysis may represent direct effects of changed ETV4 transcription, while others likely represent more indirect changes with ETV4 loss. Together, these data indicated that the repression of ETV4 played an important role in a subset of the transcriptional response to ACC1 depletion.

In order to better understand if these changes reflected an in vivo biological regulation between these genes, we developed “gene signatures” associated with the silencing of ETV4, ACC1 or ACLY using the CreateSignature algorithm [57]. These gene expression signatures represent “quantitative phenotypes” that reflect the loss of these genes. Comparing their similarity in different expression datasets allowed us to recognize similar quantitative changes in these genes in both in vitro experimental perturbation and human tumors. Similar “gene signature” approaches have been used to define the influences of oncogenic signaling and TME stresses in multiple cancer types [58–60]. Gene expression patterns from human tumor samples [61] were then separated by their similarity to our developed gene signatures associated with loss of ETV4 (shETV4), ACC1 (shACC1) and ACLY (shACLY). Binary regression from this analysis in human tumors showed highly statistically significant correlations between the shACC1 or shACLY and shETV4 signatures (Fig 4d). In other words, patient tumors with gene expression patterns more similar to the ACC1-depletion (shACC1) signature had expression patterns that were also more similar to the ETV4-depletion (shETV4) signature; likewise, patient tumors with gene expression patterns similar to the ACLY-depletion (shACLY) signature also had similar gene expression patterns with the ETV4-depletion (shETV4) signature. Importantly, this showed that the regulation between ACC1/ACLY and ETV4 was relevant in tumor expression datasets. Overall, these analyses demonstrated the similarity of the transcriptional responses to the depletion of ACC1, ACLY or ETV4 and suggested that ETV4 mediated a portion of the transcriptional effect downstream of ACLY or ACC1 both in vitro and in vivo.

### Global metabolomics revealed that hypoxia-induced elevated α-ketoglutarate levels protected cells from apoptosis

Considering that ACC1 and ACLY are critical lipogenic enzymes, we performed a metabolomics experiment to interrogate the metabolic effects of ACC1 or ACLY depletion under normoxia or hypoxia. Five cell lines were evaluated in triplicate: 1 control “hypoxia-sensitive” cell line (shScramble line) and four “hypoxia-survival” cell lines (2 shACC1 lines, 2 shACLY lines). After 36 hours of treatment, cells were lysed on ice and collected to measure the intracellular levels of 15 amino acids and 45 acyl-carnitines by tandem mass spectrometry (MS/MS) and levels of 7 organic acids by gas chromatography and mass spectrometry (GC/MS). All measurements were normalized by total protein content per sample.

**Fig 5:**
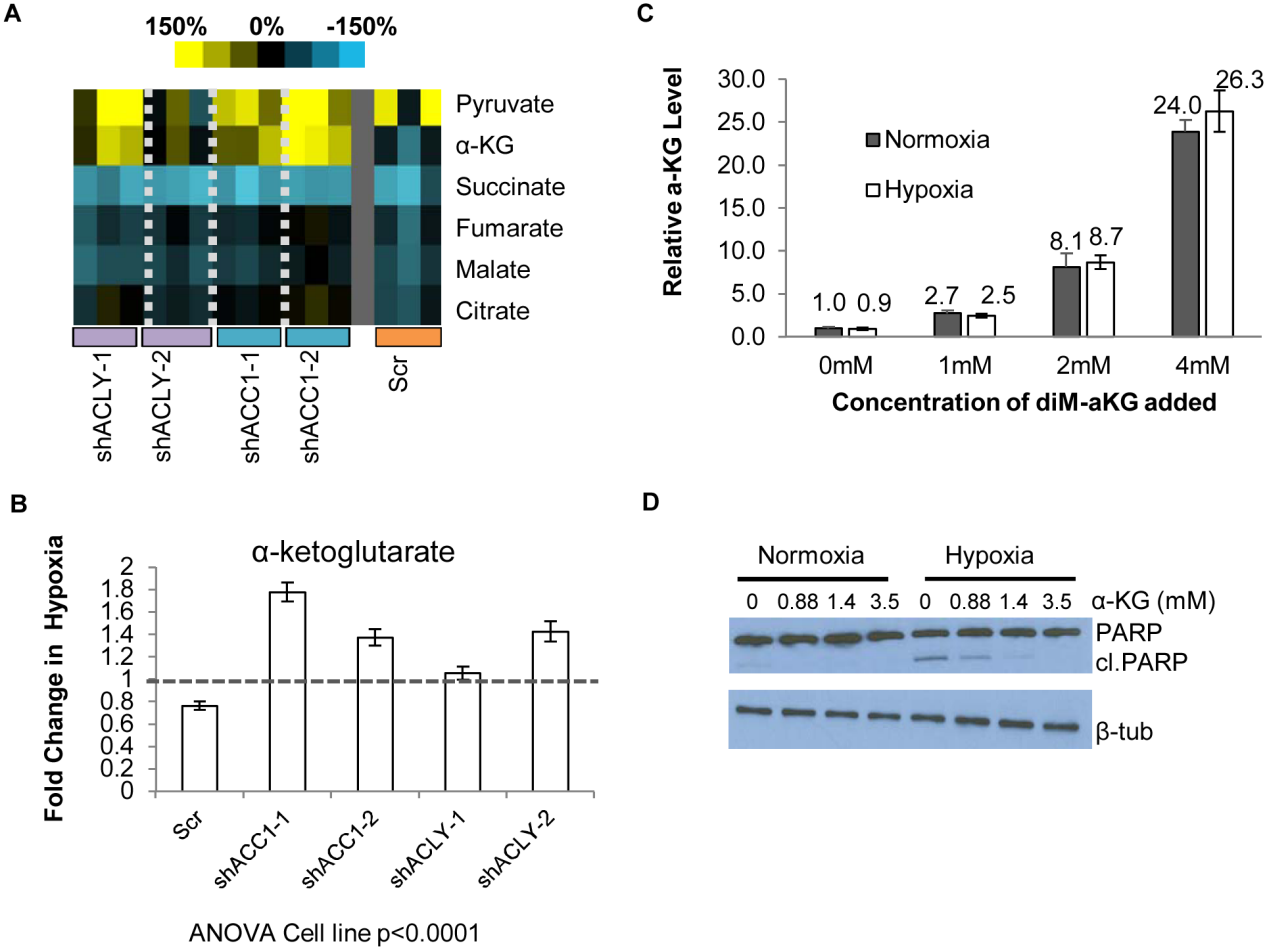
Loss of ACC1 or ACLY results in elevated levels of α-KG under hypoxia. (A) Heat map showing percent change under hypoxia of various organic acids in indicated cell lines. Yellow = increase in hypoxia; Blue = decrease in hypoxia. Each shRNA cell line (2 per gene) was done in triplicate (n = 3). (B) Protein-normalized fold change of intracellular α-KG levels in hypoxia in indicated cell lines. Dashed line indicates no change in hypoxia from normoxia (n = 3). (C) Normalized intracellular levels of α-KG after supplementation with indicated concentration dimethyl-α-KG for 24 hours (n = 3). (D) Western blot of intact and cleaved PARP after α-KG supplementation in control cells under normoxia or hypoxia for 48 hours. Data are represented as mean values +/- SEM, unless otherwise noted. Data are from the H1975 cell line.

We observed several expected metabolic changes to validate our approach. Silencing ACC1 depleted basal and hypoxia-induced palmitate levels, reflecting reduced *de novo* lipogenesis in these cells (S7a Fig). Consistent with hypoxia-induced inhibition of pyruvate utilization in the TCA cycle in favor of anaerobic glycolysis, hypoxia modestly increased the levels of pyruvate and lactate in control cells (S7b and S7c Fig). In addition, as previously noticed [62], hypoxia generally reduced the levels of TCA metabolites succinate, fumarate, malate, and citrate in most cells (Fig 5a). These results indicated that our metabolomics assay accurately detected the expected metabolic changes associated with inhibited lipogenesis and hypoxia exposure.

The pattern of α-ketoglutarate (α-KG) levels in this experiment suggested that it may be an interesting candidate for offering protection under hypoxia. In the control cells, hypoxia reduced the levels of α-KG. However, in the hypoxia-resistant cells with depleted ACC1 and ACLY, hypoxia increased the α-KG levels (Fig 5b), as has been seen before in hypoxia-resistant cells [62]. We could test the possibility that levels of α-KG contributed to survival by adding cell-permeable dimethyl-α-KG to H1975 cells. We determined the level of α-KG achieved intracellularly after extracellular supplementation to choose a supplementation treatment that would be relevant to the levels of α-KG seen with ACC1 or ACLY depletion. Mass spectrometry analysis showed increasing amounts of intracellular α-KG after supplementation in a dose-dependent manner (Fig 5c) and that dimethyl-α-KG supplementation at 1mM achieved levels of α-KG comparable to the hypoxia-induced increase found in the ACC1 and ACLY depleted cells under hypoxia (Fig 5b). Supplementation of relevant levels α-KG also inhibited PARP cleavage under hypoxia (Fig 5d). These data indicated that the increased α-KG under hypoxia in the ACC1 and ACLY depleted cells recapitulated the hypoxia-protective phenotype of these cells.

### Hypoxia-induced increased α-ketoglutarate levels regulated ETV4, possibly through 2-oxoglutarate/Fe(II)-dependent dioxygenases

**Fig 6:**
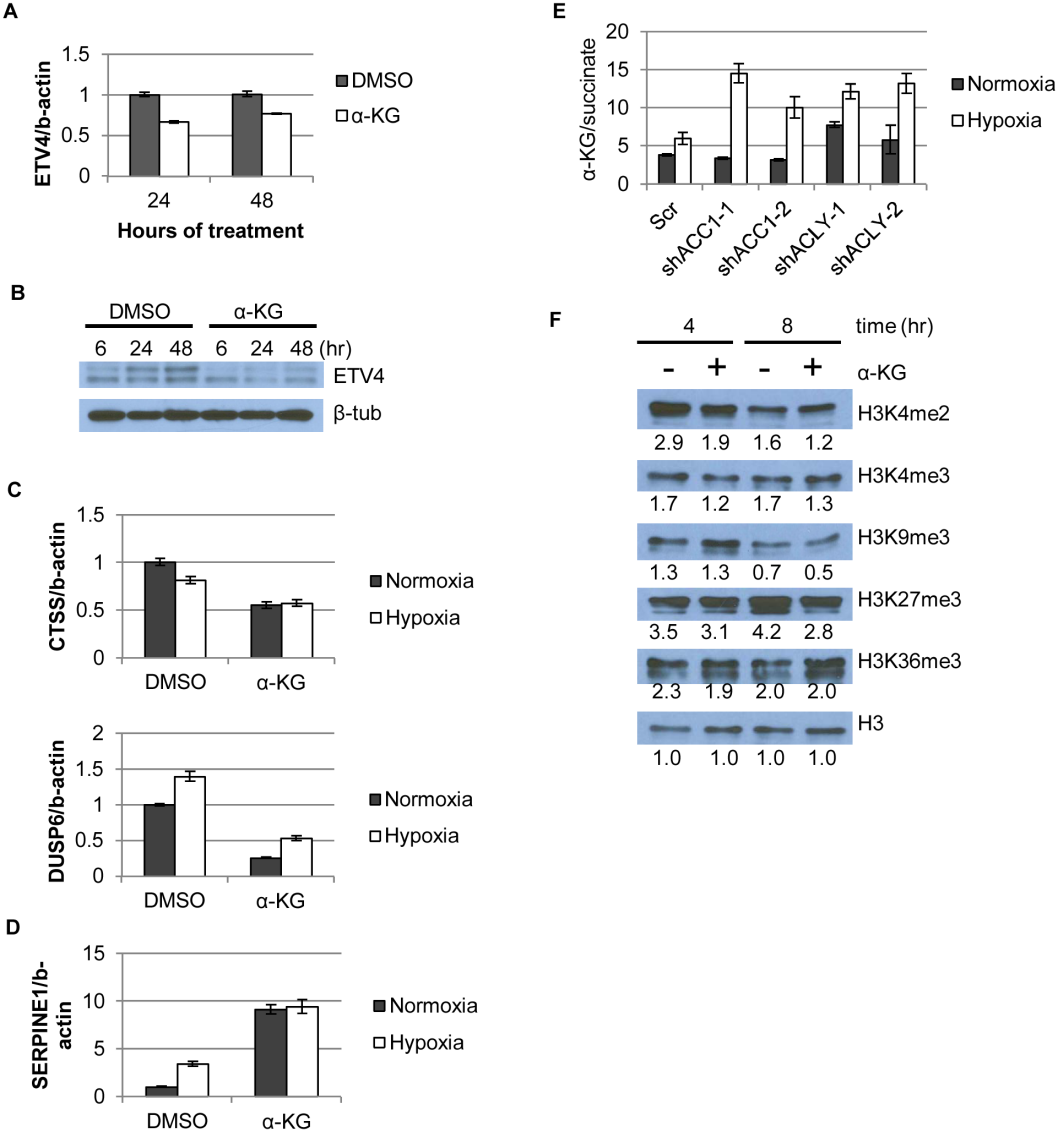
α-KG regulates ETV4 mRNA levels and activity through epigenetic changes. (A) qPCR showing ETV4 mRNA levels after α-KG supplementation (1mM) over time (n = 6). (B) Western blot showing ETV4 protein levels after α-KG supplementation (1mM) over time. (C,D) qPCR of gene expression changes after α-KG supplementation (3.5mM, 24h) for both (C) down-regulated genes (similar to Fig 4b) and (D) up-regulated genes (similar to Fig 4b and 4c) (n = 6). (E) Ratio of α-KG/succinate in normoxia and hypoxia in the indicated cell lines from the metabolomics experiment (n = 3). (F) Western blot showing effect of α-KG supplementation (1mM) on histone methylation marks after indicated time of treatment. Numbers indicate densitometry analysis by Image J relative to that sample’s loading of total H3. Data are represented as mean values +/- SEM. Data are from the H1975 cell line.

With both α-KG and ETV4 acting downstream of ACC1 or ACLY, we next determined if α-KG was mediating the effects of ACC1 or ACLY on ETV4 expression. α-KG supplementation reduced mRNA and protein levels of ETV4 at both lower (Fig 6a and 6b) and higher concentrations (S7d and S7e Fig). Additionally, α-KG supplementation in control cells caused similar changes in repressed and induced genes as was caused by ACC1, ACLY or ETV4 silencing (Fig 6c and 6d), and some of these mRNA effects were dose-dependent with α-KG supplementation (S7f and S7g Fig). Unexpectedly, diM-α-KG supplementation increased HIF–1α protein levels (S7h Fig). Since the regulation of HIF–1α was different with depletion of ACC1/ACLY or α-KG supplementation, the changes in HIF–1α protein did not likely explain the improved hypoxic cell survival of the ACC1/ACLY depleted cells. Combined, these data indicated that, in the ACLY or ACC1 depleted cells, the α-KG increase was a hypoxic trigger that reduced ETV4 levels and activity to mediate an anti-apoptotic gene expression response.

In addition to being a TCA cycle intermediate, α-KG is a substrate for the abundant 2-oxoglutarate/Fe(II)-dependent dioxygenases (2-OGDDs) [26]. 2-OGDDs use α-KG and molecular oxygen as substrates to perform a number of different protein modification reactions. These enzymes include families of histone demethylases that recognize and remove methylation marks from histones, as well as the TET family of proteins that facilitate DNA demethylation [26]. Thus, α-KG levels can affect gene expression through the activities of these 2-OGDDs [28]. An elevated ratio of α-KG/succinate (substrate/product ratio) has been proposed as a potential indicator of increased 2-OGDD activity [25,28]. We found that the α-KG/succinate ratio was significantly elevated in all of the shACC1 and shACLY cells under hypoxia (Fig 6e). To better understand if the level of α-KG or the α-KG/succinate ratio determined our hypoxia-survival phenotypes, we supplemented ACLY or ACC1 depleted cells with cell-permeable dimethyl-succinate to theoretically drive the α-KG/succinate ratio in the opposite direction from when α-KG was added. Succinate supplementation did not affect the survival of either shACC1 or shACLY cells under hypoxia (S8a and S8b Fig) and also did not affect the regulation of ETV4 by ACC1 or ACLY (S8c and S8d Fig). Therefore, in our experimental system, we concluded that the levels of α-KG, rather than the α-KG/succinate ratio, were driving the hypoxia survival phenotypes.

While the succinate supplementation did not affect our phenotype, the elevated α-KG in the ACC1 or ACLY depleted cells under hypoxia still suggested that 2-OGDDs may be relevant in our system. Therefore, we hypothesized that α-KG affected ETV4 mRNA abundance by altering the activity of 2-OGDDs and subsequent histone methylations. As a control, we tested the DNA methylation status of the two shore regions and the center region of the ETV4 promoter CpG islands by bisulfite pyro-sequencing and saw no significant change upon α-KG supplementation (S8e Fig). However, α-KG caused a global reduction in two (H3K4me2 and H3K4me3) “active” histone methylation marks and also globally reduced two “repressive” marks, H3K27me3 and, to a lesser extent, H3K9me3 (Fig 6f). When we compared the epigenetic changes associated with α-KG supplementation with Carey et al.[28], we found that H3K27me3 was consistently reduced with the addition of α-KG in both studies. However, there were also differences in the histone methylation changes caused by α-KG across the studies: 1) H4K20me3 was reduced with α-KG previously and we saw an increase in this mark (S8f Fig); 2) whereas we saw a decrease in the levels of H3K4me3, no changes were seen previously. These differences could be due to different cellular contexts (embryonic stem cells vs. cancer cells) or the presence of glutamine deprivation during the previous examination of α-KG effects [28]. Similar changes were observed using either water or DMSO as control (S8g Fig). Collectively, the reduced levels of multiple methylation marks were consistent with our hypothesis that predicted more active histone demethylases as a result of increased levels of α-KG.

**Fig 7:**
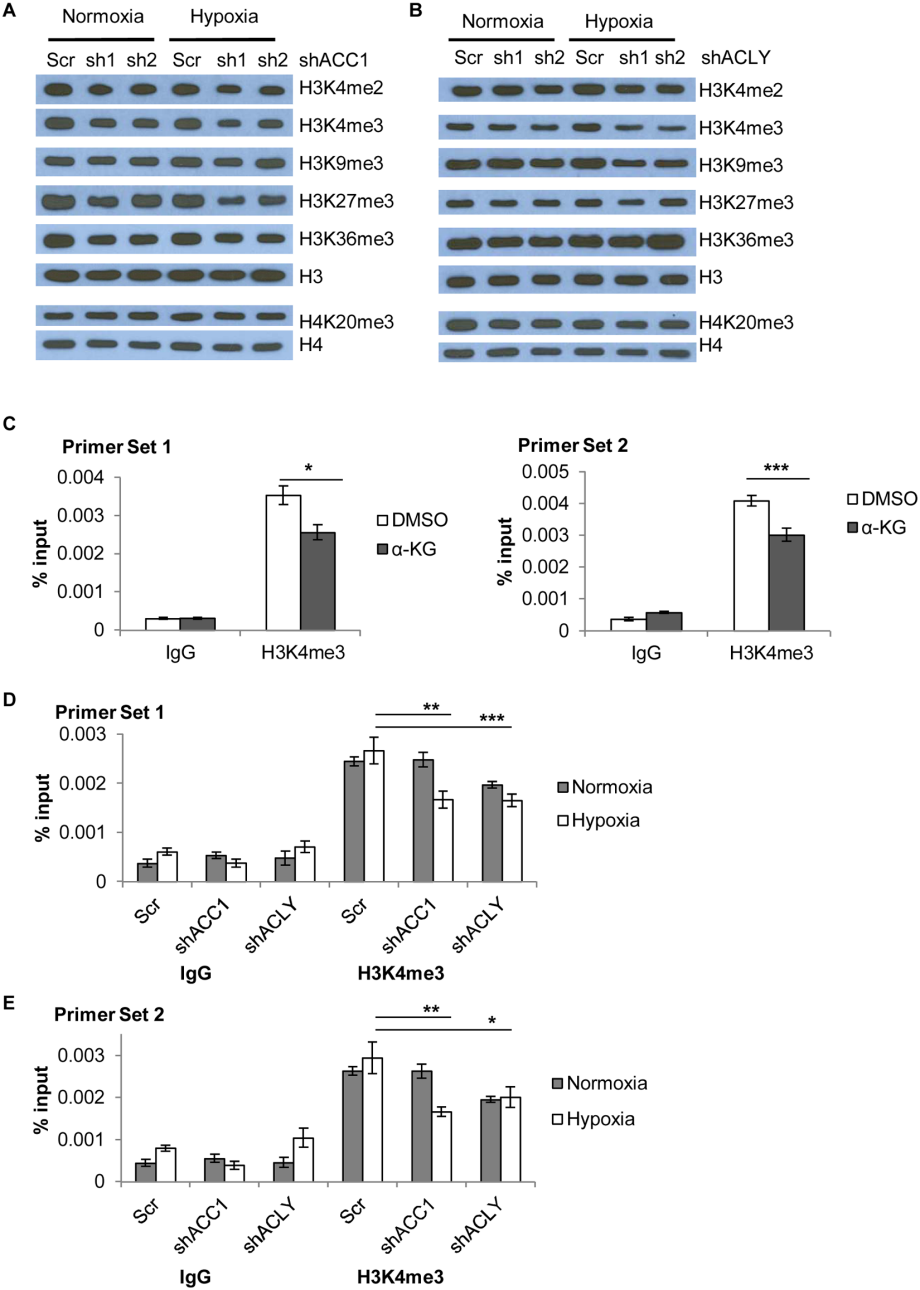
Histone methylation changes in shACC1 and shACLY cells under hypoxia. (A) Western blot showing levels of the indicated histone methylation marks in shACC1 cells under normoxia or hypoxia (24h). (B) Western blot showing levels of the indicated histone methylation marks in shACLY cells under normoxia or hypoxia (8h). (C) ChIP-qPCR analysis of the abundance of the H3K4me3 mark at two locations in the promoter region of ETV4 after α-KG treatment (n = 6). (D, E) ChIP-qPCR analysis of the abundance of the H3K4me3 mark at two locations in the promoter region of ETV4 after ACC1 or ACLY depletion under normoxia and hypoxia (n = 9). n.s. = not significant; * = p<0.05, ** = p<0.01, *** = p<0.005, ***** = p<0.0001. Data are represented as mean values +/- SEM. Data are from the H1975 cell line.

To extend evidence in support of our model, we also examined various histone methylation modifications associated with the depletion of ACC1 or ACLY. Overall, we saw similar global histone methylation changes in both the shACC1 (Fig 7a) and shACLY (Fig 7b) cells as compared to α-KG supplementation. Among all the tested epigenetic markers, the H3K4me3 mark was the most pronouncedly decreased across both gene depletions and the α-KG treatment. The H3K4me2 mark was decreased in all three conditions (shACC1, shACLY, α-KG supplementation) to a modest degree. Similarly, H3K9me3 was decreased somewhat with the 8 hour α-KG treatment and in the shACC1 cells, while it was more strongly decreased in the shACLY cells. H3K27me3 was lowered by both α-KG and ACC1 depletion. Levels of H4K20me3 were unchanged in the shACC1 and shACLY cells while they were increased with α-KG treatment, and so this suggested that the changes in this methyl mark were likely not due to the changes we explain in our model.

Besides global epigenetic changes, we also determined if histone methylation at the ETV4 locus was changed by chromatin immunoprecipitation of the “active” histone H3 lysine 4 tri-methylation (H3K4me3) mark. This mark was chosen because it showed the most consistent and strongest changes across either genetic depletion or with α-KG supplementation. In addition, a loss of this “active” mark H3K4me3 would be consistent with decreased ETV4 expression in these conditions. ChIP experiments showed that α-KG treatment decreased the abundance of the H3K4me3 modification at the ETV4 locus by ~30% (Fig 7c). Additionally, there was a decreased abundance of H3K4me3 in both ACC1 and ACLY depleted cells under hypoxia as compared to control cells (Fig 7d and 7e). Together, these data showed that elevated levels of α-KG affected the histone, but not DNA, methylation status of the ETV4 locus and this pattern was similar to the histone methylation changes seen under hypoxia in the shACC1 and shACLY cells.

**Fig 8:**
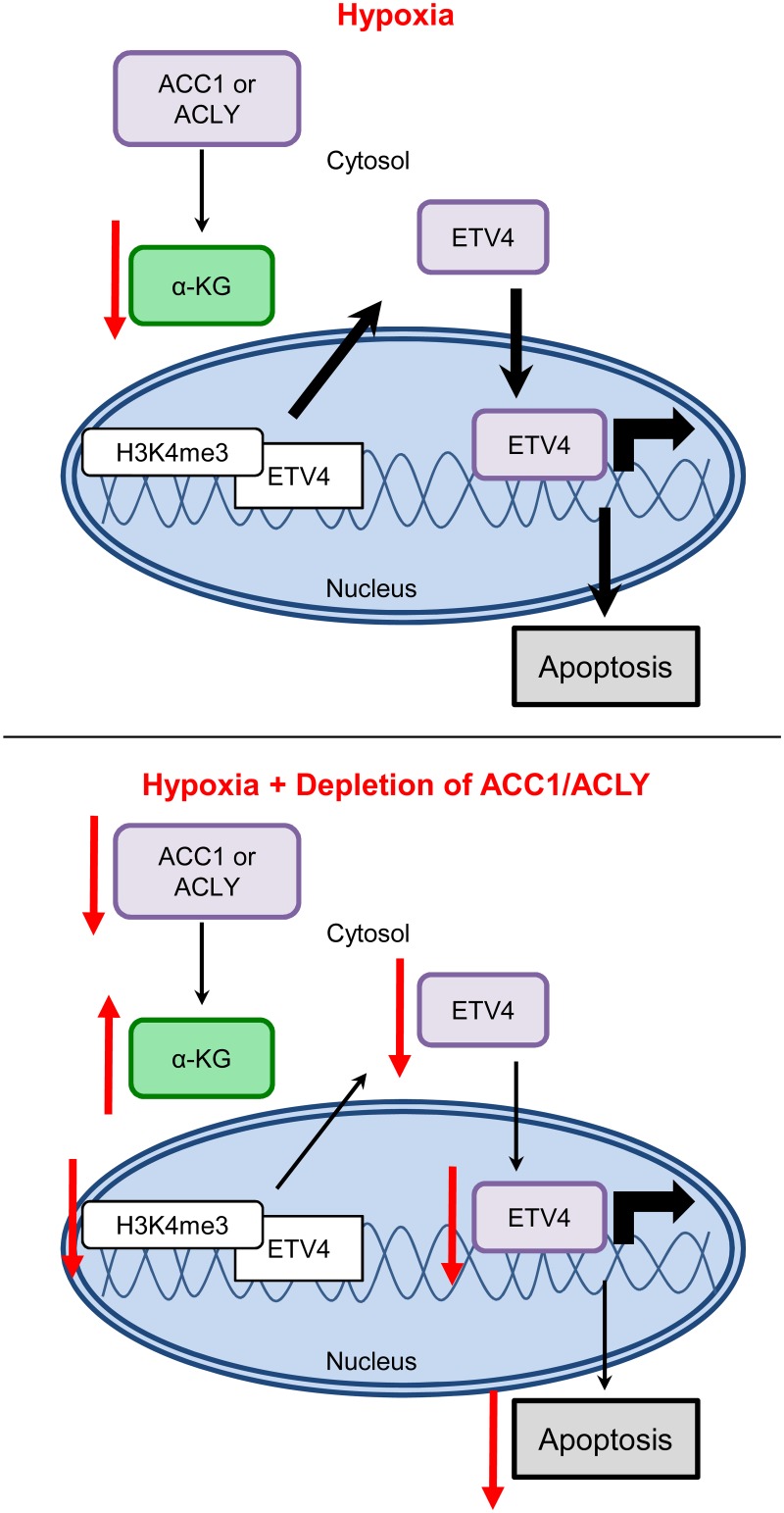
Model for how loss of ACLY, ACC1, or ETV4 protects cells from hypoxia-induced apoptosis.

Collectively, our data was consistent with a model in which, under hypoxia, the inhibition of ACC1 or ACLY increases levels of α-ketoglutarate to block hypoxia-induced apoptosis by reducing the levels and activity of ETV4, possibly through altered histone methylation patterns (Fig 8). These data offer a novel molecular connection showing that the transcriptional output of altered lipogenic metabolism can modulate the cellular response to and survival under hypoxia.

## Discussion

Here we have described a pooled shRNA screen that successfully identified genes that influence cancer cell survival under hypoxia and lactic acidosis. Specifically, we showed that blocking *de novo* lipogenesis through the genetic depletion of *ACLY* or *ACC1* protected multiple cancer cells from hypoxia-induced apoptosis through increased levels of α-ketoglutarate and the inhibition of ETV4 and its transcriptional activities. Therefore, inhibition of ACLY or ACC1 affected both metabolism and transcription to protect cells from hypoxia-induced apoptosis. These results also suggest that one important mechanism of hypoxia-induced apoptosis is through the reduction of α-KG, which potentially elevates the levels and activity of ETV4 through histone modifications to promote oncogenesis and trigger apoptosis.

Inhibitors that target ACLY and ACC1 are proposed cancer therapeutics [63,64]. ACC1 has been targeted as it is the rate-limiting enzyme in lipogenesis, a process critical for cancer cells’ rapid proliferation [63]. For ACLY, not only is it a critical enzyme for active lipogenesis, but it also regulates epigenetic states by generating acetyl-CoA, and so it offers two mechanisms to target clinically [20]. Our data provides several insights on the biological effects of ACC1 and ACLY in hypoxic cancer cells that should be considered when targeting these enzymes. First, while we expect ACLY and ACC1 inhibition to have opposite effects on levels of acetyl-CoA, their effects on gene expression were highly similar. This suggests that levels of acetyl-CoA (and subsequent histone acetylation or changes in epigenetic states) may not readily explain the majority of gene expression responses to the inhibition of ACC1 or ACLY under hypoxia. Instead, the ACC1/ACLY-induced reduction in ETV4 levels and activity seemed to account for a significant portion of the hypoxic transcriptional changes. These results suggest that lipogenic inhibitors that block ACC1 or ACLY may be particularly effective for tumors driven by *ETV4*. Likewise, this reduction of the oncogenic driver ETV4 may account for portions of the therapeutic potential of lipogenic inhibitors. However, our data also showed that these treatments may allow for the survival of slowly proliferating cancer cells in regions of hypoxic tumors. Therefore, targeting lipogenesis in cancer may need to be combined with other therapeutic approaches that target hypoxic regions (such as hypoxia-activated pro-drugs), to eliminate the cancer cells that may be dormant and protected in the hypoxia-niche.

Elevated α-KG levels in the shACLY and shACC1 cells under hypoxia could be due to increased generation or decreased consumption of this metabolite. A portion of α-KG is consumed during lipogenesis, which is further promoted under hypoxia by reductive carboxylation [12,65]. Therefore, blocking lipogenesis under hypoxia may lead to a “build-up” of upstream metabolites, including α-KG. It is well appreciated that α-KG plays a critical role in supporting cell survival by replenishing the metabolic intermediates of the TCA cycle. Here, our data indicated than an additional manner by which α-KG can affect cellular survival under hypoxia was by regulating ETV4 expression, possibly through epigenetic mechanisms.

Transcriptional adaptation to hypoxia is most often orchestrated by the HIFs; however, here we showed that cancer cells’ hypoxic survival can be mediated by a different transcription factor, ETV4. ETV4 was proposed as an essential co-activator of HIF–1α and to have a hypoxic transcriptional program [66]. Our data revealed that ETV4 was critical for hypoxia-induced apoptosis. Interestingly, the levels of ACC1 or ACLY influenced α-KG and ETV4 levels under hypoxia, thereby providing a link between lipogenesis, a TCA cycle intermediate and transcription. Therefore, ETV4 mediated the transcriptional output of varying degrees of active lipogenesis caused by changing ACC1 and ACLY levels. Our gene signature analyses also suggested that this regulation was preserved in human tumors in vivo. While the global and local ETV4 epigenetic changes we describe herein were consistent with decreased activity of ETV4 to protect cells from hypoxia-induced apoptosis, our evidence provides only correlative support with reduced promoter activity and does not provide a causal explanation for the ETV4 repression. Additionally, although this regulation seemed mostly specific to ETV4, we do not fully exclude the possibility that other ETS transcription factor family members contribute to an apoptotic, hypoxic transcriptional program. Overall, our data establishes ETV4 as one critical factor that influences hypoxic cell survival and transcriptional responses downstream from ACC1 and ACLY, and reveals the metabolite α-ketoglutarate as a molecular link between metabolic and transcriptional adaptation to hypoxia.

In addition to its importance in the TCA cycle, our experiments showed that elevated α-KG could alter histone methylation patterns, likely via α-ketoglutarate-dependent dioxygenases, to potentially regulate ETV4. While the causal relationship of several regulatory steps of this hypothesis was not rigorously tested by genetic manipulations here, our data was consistent with such a model. Previous reports suggested that α-KG/succinate ratios determined the direction and activities of dioxygenases [28], yet our data indicated that it was increased α-KG, not succinate or α-KG/succinate, that drove the epigenetic changes, ETV4 repression and hypoxia survival phenotypes. As a substrate, α-KG levels likely affect the enzymatic activities of of the 2-OGDDs histone demethylases, each of which have different sets of lysine residues upon which they act [26]. The number and combination of global histone methylation events that were changed with depletion of ACC1, ACLY or α-KG treatment suggested that many different demethylases could be affected by α-KG or the depletion of ACC1/ACLY in these cellular states. While there are no histone methyltransferases currently known to use α-KG as a substrate, they are regulated by hypoxia [67,68], and so we do not exclude the potential for changes in histone methylation patterns to be due to changes in both methyltransferase and demethylase enzyme activities. Additionally, the changes in global histone methylation patterns are very likely affecting a number of other genes’ expression patterns, in addition to the changes we see at the ETV4 locus.

Since α-ketoglutarate supplementation and loss of ACC1 or ACLY reduced the abundance of H3K4me3 at the ETV4 promoter, we speculate that the JARID1 (KDM5) family of histone demethylases, which specifically demethylate H3K4me2/3 residues, could be affecting the abundance of this mark at the ETV4 locus [69,70]. These enzymes are known to range in their expression and activity by cell type and are differentially influenced by oxygen levels [71–73]. A full investigation in to the mRNA, protein and activity levels of many of these family members would be necessary to determine the extent to which each plays a role in regulating ETV4 under hypoxia. Effort has been made to investigate the JARID1 family of H3K4 demethylases as potential cancer drug targets [70,74,75] as the importance of a mis-regulated “histone code” for tumorigenesis is well recognized. While some reports suggest a tumor suppressive role of these enzymes, more suggest an oncogenic function [69]. This information and the data we presented here suggest that various 2-OGDDs in distinctive contexts differentially affect tumorigenesis and tumor cell survival. It will be important to understand the proper context of treatment if these drugs continue into the clinic.

Multiple previous reports show that the activity of ACLY [64,76,77], ACC1 [78–81] or ETV4 [82–85] is associated with increased tumorigenicity and/or poor patient outcome, or that inhibiting these genes’ activities reduces tumorigenicity and improves patient outcome. We show that the inhibition of ACLY, ACC1 or ETV4 paradoxically allows tumor cells to survive better under hypoxia. To address this apparent conundrum, we propose a conceptual model in which there are two cellular states: one of activating oncogenesis and the other of stress survival. This model includes a trade-off between the two states, such that the promotion of one comes at the expense of the other. Stated another way, the activation of oncogenic programs may also render cells susceptible to apoptosis, especially under stress; likewise, reduced oncogenic programs slow cellular proliferation to a “dormant” state that could allow for better stress survival. A similar model has been proposed for several oncogenes. For example, oncogenesis driven by MYC rendered non-transformed cells vulnerable to hypoxia-induced apoptosis [86,87]; the degradation or cleavage of c-MYC under hypoxia allowed tumor cells to evade hypoxia-induced apoptosis [88,89]. E2F can promote proliferation (oncogenesis) or apoptosis in different contexts, such as with differing PI3K activity [90]. A recent paper also indicated that HIF–1α repressed the ATF4 stress response pathway to allow for the expansion of fetal cardiomyocytes [91]. According to this proposed model and our data, cancer cells treated with ACLY or ACC1 inhibitors (including metformin), may die due to blocked lipogenesis, but may also survive in hypoxic regions. As these cells resist death under stress, they may become the “dormant” cells that recur after treatment regimens end, but they also may become more targetable as they persist under hypoxia. Consistent with past literature and clinical attempts to target these genes, our model advises that treatment regimens be carefully considered. While data to prove such a model remains necessary, this study suggests that the ACLY-ACC1-ETV4 axis might mediate the balance between oncogenesis and stress survival under hypoxia. It is very likely that similar mechanisms of balance between proliferation and stress survival exist in a wide variety of biological contexts.

## Materials and Methods

### shRNA screen and analysis

H1975 cells were spin-infected with the pMSCV-based retroviral genome-wide library, at an MOI of 0.3, divided into six sub-pools, achieving a final library representation of 1000 cells per shRNA after selection with 1 ug/ml puromycin [37]. After three days of puromycin selection, cells were split into control and stress conditions, maintaining 1000-fold representation of each shRNA per triplicate. Cells were serum starved to 0.1% FBS 24 hours after plating. 24 hours after serum starvation, media was changed to treatment media (0.1% FBS, 25mM Hepes); control and hypoxia media pH = 7.4; lactic acidosis treatment had 25mM lactic acid (Sigma cat. no L6402) adjusted to pH = 6.7 and filter sterilized. After 4 days of treatment, cells were harvested, centrifuged, and frozen at -80°C. Genomic DNA was extracted with the QIAamp DNA Blood Maxi kit (QIAGEN, cat.no 51194) then shRNA sequences were PCR amplified. The amplified products from the control and each stress were labeled (Cy3 and Cy5, respectively) and then interrogated by a custom Agilent microarray, which contained probes against the library’s shRNA sequences [37]. We validated the sensitivity and specificity of the array to different ratios of labeled PCR product (S1a Fig). Therefore, relative hybridization of the Cy5/Cy3 labeled shRNA populations determined the abundance of each shRNA under control, hypoxia or LA. The Cy3 and Cy5 signals across the three biological replicates were highly reproducible (S1b Fig). Probes with signal intensities of less than 2-fold above background were discarded. Cy5/Cy3 ratios, also called “R/G” ratios, for remaining probes were calculated, log2 transformed and quantile normalized across pools. The R/G ratios ranged from +/- 4.0, although many fell in the “unchanged” range of +/-0.5 (S1c Fig).

For the “multiple hairpin analysis,” genes were considered a hit when they had 1) at least 2 different shRNAs with (absolute value R/G) > 0.7 in at least 2 of the three biological replicates (2) the (stdev/ave) of the biological replicates was <0.5 (S1 Table).

### Cell culture, TME stress treatments and generation of stable shRNA cell lines

H1975 cells were cultured in RPMI media (GIBCO cat. no 11875) supplemented with 10% Fetal bovine serum (heat-inactivated), 1% glucose, 10mM HEPES, 1mM sodium pyruvate, and 1x antibiotics (penicillin, 10,000 UI/ml; streptomycin, 10,000 UI/ml), as directed by the Duke Cell Culture Facility. MDA-MB–231 and PANC–1 cells were cultured in DMEM (GIBCO cat.no. 11995) supplemented with 10% Fetal bovine serum (heat-inactivated) and 1x antibiotics (penicillin, 10,000 UI/ml; streptomycin, 10,000 UI/ml). Cell lines, obtained from and initially validated by the Duke Cell Culture Facility (Durham, NC, USA), were maintained for fewer than 6 months and validated by microscopy every 1 to 2 days.

Lactic acidosis was generated via addition of lactic acid (Sigma-Aldrich, St. Louis, MO, USA, cat. no L6402) and media pH adjustment to pH 6.7 by HCl immediately before use. Hypoxia was generated with a cell culture incubator with 93–94% N_2_, 5% CO and 1–2% O_2_. For the α-KG rescue experiments, media was supplemented with 0.875-4mM dimethyl α-KG as indicated in figure legends (Sigma, cat. no. 349631). For all stress experiments, cells were serum starved (0.5% FBS) for 24 hours before treated with stress under 0.5% FBS. All survival/viability measurements were made after 4 days of stress treatment.

### Stable cell line generation

Stable cell lines were created with the pLKO.1 shRNA constructs purchased from the Duke RNAi Core Facility. Virus was generated by transfecting HEK-293T cells with a 1: 0.1: 1 ratio of pMDG2: pVSVG: pLKO.1 with Lipofectamine 2000 in the evening. Media was changed the following morning and virus collected 48 hours after transfection. Stable cell lines were generated by adding 200ul virus to a 60mm dish of parental cells with polybrene (final concentration 8ug/ml). Complete death in blank infection dishes was used to determine success of infection and puromycin selection. The efficiency of silencing or overexpression was determined by western blots. Concentrations of puromycin needed for selection: H1975 cells = 1ug/ml, MDA-MB–231 cells = 1ug/ml, PANC–1 cells = 2ug/ml. For stable overexpression, concentration of blasticidin used was 2.5ug/ml in H1975 cells.

### Crystal violet staining

Cells were fixed either in 4% paraformaldehyde (PFA) overnight at 4°C or at room temperature for 30 min. PFA was removed and crystal violet staining solution (0.2% crystal violet, 25% methanol, 75% water) gently shaken on cells for 30+ minutes at room temperature. Staining solution was removed and plates rinsed with tap water 2–3 times. For quantitation, completely dried stain was dissolved by adding 10% acetic acid and shaking gently at room temperature for 30+ min before reading absorbance at 570 nm.

### Determination of cell number

Cell number was evaluated by either direct cell counting (trypan blue exclusion) or high-throughput microscopic counting (HTC) of fixed and stained nuclei. For direct cell counting, at designated time after treatment, media was removed, cells were not rinsed for fear of losing loosely-attached cells, trypsinized, diluted 1:1 with trypan blue and immediate counted on a hemocytometer. For HTC experiments, after designated time period, cells were fixed in 4% PFA either overnight at 4°C or for 30 min at RT. Cells were washed 2x, permeabilized with 0.1% Triton-X in PBS, wash 2x, stained with 50ug/ml Hoescht dye (Sigma cat. no B2261) for 30 min, RT in the dark, then washed 2x and PBST added to each well and scanned by the Cellomics high-throughput microscope at the Duke RNAi Core Facility.

### Flow cytometry

For cell cycle analysis, after 4 days of stress treatment, media was collected, cells trypsinized and pooled with the media. Cells were centrifuged then fixed by resuspension in ice cold 70% ethanol while gently vortexing. Fixed cells were placed at -20°C until prepared for FACS analysis. Immediately before FACS analysis, cells were centrifuged for 5 min at room temperature, washed twice in PBS then resuspended in 25ug/ml Propidium iodide (Sigma cat. no P4864) and 10ug/ml RNAse A in PBS. Cells were stained for 30+ min in the dark then 8000 events measured on a Canto II Flow cytometer.

### Protein lysate collection and Western blots

Cell lysis: Cells were washed once with ice cold PBS, lysed by RIPA buffer with protease and phosphatase inhibitors added fresh, scraped into a microcentrifuge tube, allowed to swell on ice for 15–20 min, vortexed briefly, then spun down at top speed for 15 min at 4°C. Supernatant was transferred to pre-cooled new tube and protein concentration assayed with the Pierce BCA kit (ThermoScientific, cat. no. 23225). Effort was made to immediately rinse and lyse cells coming from a hypoxia condition as cells very quickly re-equilibrate to normoxic conditions. Western blots: Between 15-30ug of lysate was loaded on SDS-PAGE gels, wet-transferred to PDVF membrane, blocked with 5% milk in 1xTBST (0.1% Tween–20), then primary antibodies were incubated overnight at 4°C. For analysis of histones, protein was extracted with the EpiQuick Total Histone Extraction Kit (Epigentek, cat.no. OP–0006) and 2ug of protein were resolved on 15% SDS-PAGE gels or nuclear fractions were collected by the REAP fractionation method [92] and 7.5–30 ul of lysate were run in each lane. Please contact for details on antibody usage.

### Quantitative real-time PCR

RNA was extracted using the RNeasy Kit (QIAGEN). A total of 1 μg of total RNA was reverse transcribed by SuperScript II (Invitrogen) for real-time PCR with Power SYBRGreen Mix (Applied Biosystems/Life Technologies (Grand Island, NY, USA)). Primers were designed across exons whenever possible, verified for specificity by regular PCR prior to use in real-time PCR. Please contact for the sequences of primers used.

### Microarrays and analysis

Samples were collected on ice and RNA was isolated with QIAGEN’s RNeasy Mini Kit (cat. no 74104) according to manufacturer’s instructions. After quality control assessment with the Agilent BioAnalyzer, cDNA was amplified from 200ng RNA with the Ambion MessageAmp Premier RNA Amplification (Life Technologies, Grand Island NY, USA). The gene expression pattern of the RNA samples were interrogated with Affymetrix U133A genechips and normalized by the RMA (Robust Multi-Array) algorithm. cDNA synthesis and microarray interrogation was performed by the Duke Microarray Core. The influence of the silencing of *ACC1* or *ACLY* on gene expression was derived by a zero transformation process, in which we compared transcript level for each gene in cells with stably integrated shRNAs targeting ACC1 or ACLY to the average transcript levels in control scramble shRNA cell line samples. Data was then filtered as described with Cluster 3.0 software and heat maps were generated with TreeView. To generate gene signatures of knockdown of ACLY, ACC1 or ETV4, the CreateSignature module in GenePattern (https://genepattern.uth.tmc.edu/gp/pages/login.jsf) was used with scramble cells expression pattern as the train0 set, knockdown cells’ gene expression pattern as train1 set and the Gray dataset [61] used as the test set. Default parameters were used for the analysis similar to [58]. The resulting probabilities of gene signature expression in each patient for each knockdown signature were analyzed by simple linear regression in the JMP Pro 11 software.

### Statistical analysis

Data, unless otherwise noted, represent the mean +/- the standard error of the mean and n indicates number of replicates used to generate the SEM. P-values were determined either by a two-tailed Student’s t-test in Excel or by a two-way ANOVA with StatView.

### Metabolomics profiling and analysis

The measurement of amino acids and acyl carnitines was performed using stable isotope dilution techniques and flow injection tandem mass spectrometry and mass spec sample preparation methods described previously [93–95]. Derivatized organic acids were analyzed by capillary gas chromatography/mass spectrometry (GC/MS) using a TRACE DSQ instrument (Thermo Electron Corporation; Austin, TX) [93–95]. All MS analyses employed stable-isotope-dilution. The standards serve both to help identify each of the analyte peaks and provide the reference for quantifying their levels. Quantification was facilitated by addition of mixtures of known quantities of stable-isotope internal standards from Isotec (St. Louis, MO), Cambridge Isotope Laboratories (Andover, MA), and CDN Isotopes (Pointe-Claire, Quebec, CN) to samples. Sample Preparation: Biological triplicates of 15cm plates under each treatment condition were placed on ice and washed twice with ice-cold PBS before as much PBS as possible was removed. Cells were lysed in 620ul ml of 0.78% Formic Acid in water and scraped to collect. 30ul were removed for protein quantification. 1x volume of the collected pellet (~800ul) of acetonitrile was added and sample was vortexed vigorously. Aliquots were separated for mass spectrometry measurements (300 ul for organic acids, 100 ul for amino acids/acyl-carnitines) and were immediately frozen on dry ice and transferred to -80°C. Analysis: Protein concentration per replicate was determined by the Pierce BCA Kit (ThermoScientific, Waltham, MA, USA) and used to normalize all metabolite levels.

### NADP+/NADPH measurements

A ratio of NADP+/NADPH was calculated after measuring each molecule separately with the Amplite Fluorimetric NADP/NADPH Ratio Assay Kit from AAT Bioquest, Inc (Sunnyvale, CA). Protocol was conducted as the manufacturer suggested and all values were normalized to protein content, as measured by the Pierce BCA kit, on similarly plated and treated samples done in parallel.

### Chromatin Immunoprecipitation

3 million H1975 cells were plated in 15 cm dishes, after 24 hours they were serum starved to 0.5% FBS, 24 hours later they were treated with either a 4-hour treatment of α-KG or an 8 hour treatment of hypoxia before collection for a native ChIP. Protocol was carried out as manufacturer suggested with the SimpleChIP Plus Enzymatic Chromatin IP Kit (Agarose Beads) (Cell Signaling Tech., cat. no. 9004). Sonication and digestion were performed to obtain chromatin 1–4 nucleosomes in size, which was verified by gel electrophoresis. Each IP used chromatin prepared from 4–5 million cells and was performed overnight.

### DNA Methylation

Genomic DNA was extracted with the DNeasy Blood & Tissue Kit according to the protocol provided by the manufacturer (Qiagen). The genomic DNAs (800 ng) were modified by treatment with sodium bisulfite using the Zymo EZ DNA Methylation kit (Zymo Research, Irvine, CA). Bisulfite treatment of denatured DNA converts all unmethylated cytosines to uracils, leaving methylated cytosines unchanged, allowing for quantitative measurement of cytosine methylation status. Pyrosequencing was performed using a Pyromark Q96 MD pyrosequencer (Qiagen). The bisulfite pyrosequencing assays were used to quantitatively measure the level of methylation at CpG sites contained. Assays were designed to query CpG islands using the Pyromark Assay Design Software (Qiagen). Pyrosequencing was performed using the sequencing primer. PCR conditions were optimized to produce a single, robust amplification product. Defined mixtures of fully methylated and unmethylated control DNAs were used to show a linear increase in detection of methylation values as the level of input DNA methylation increased (Pearson r > 0.98 for all regions). Once optimal conditions were defined, each assay was analyzed using the same amount of input DNA from each specimen (40 ng, assuming complete recovery after bisulfite modification). Percent methylation for each CpG cytosine was determined using Pyro Q-CpG Software (Qiagen).

## Supporting Information

S1 Fig Quality control analysis of shRNA microarray and screens. (A) Correlation plots showing PCR products, when mixed at different indicated ratios, are distinguishable by the custom microarray. (B) 3-D scatterplots showing reproducibility between biological triplicates’ of hypoxia and lactic acidosis treated samples for both Cy3 and Cy5 signals. (C) Distribution of R/G ratios by number of shRNAs, separated by treatment (LA = lactic acidosis, H = hypoxia) and replicate (n = 3).
(TIF)

S2 Fig Depletion of ACLY or ACC1 is protective under hypoxia in different cell types. (A) Western blot of ACC1 protein knockdown by 2 shRNAs in MDA-MB–231 cells. (B) Quantified crystal violet of shACC1 MDA-MB–231 cells after 4 days of hypoxia (n = 3). (C) Western blot of ACLY protein knockdown by 2 shRNAs in MDA-MB–231 cells. (D) Quantified crystal violet of shACLY MDA-MB–231 cells after 4 days of hypoxia (n = 3). (E) Western blot of ACC1 protein knockdown in PANC–1 cells. (F) Western blot of ACLY protein knockdown in PANC–1 cells. (G) Quantified crystal violet staining of indicated shRNA PANC–1 cells after 6 days of hypoxia (n = 3). (H) Crystal violet staining of H1975 cells with simultaneous metformin treatment and hypoxia for 4 days. (I) Western blot of PARP in H1975 cells with hypoxia and metformin treatment. (J) Counts of viable cell number by trypan blue exclusion of shACC2 cells under normoxia or hypoxia for 4 days (n = 9). (K) and (L) Crystal violet of ACC1 and scramble (Scr) cells (boxed in blue rectangles) under indicated stresses (K-hypoxia, L- LA (lactic acidosis), no glutamine or no glucose (Glu)) for 4 days. Data are represented as mean values +/- SEM.
(TIF)

S3 Fig Depletion of ACLY or ACC1 leads to decrease HIF–1α protein expression in multiple cell types. (A) Western blot of HIF–1α protein levels with ACC1 knockdown by 2 shRNAs in H1975 cells. (B) Western blot of HIF–1α protein levels with ACC1 knockdown by 2 shRNAs in MDA-MB–231 cells. (C) Western blot of HIF–1α protein levels with ACC1 knockdown by 2 shRNAs in PANC–1 cells. (D) Western blot of HIF–1α protein levels with ACLY knockdown by 2 shRNAs in H1975 cells. (E) Western blot of HIF–1α protein levels with ACLY knockdown by 2 shRNAs in MDA-MB–231 cells.
(TIF)

S4 Fig Depletion of ACLY or ACC1 does not protect through NADPH, ATP or other PEA3 family members. (A) NADP+/NADPH ratio under normoxia and hypoxia in shScr or shACC1 H1975 cells (n = 6). (B) Crystal violet staining of shScramble H1975 cells treated with N-acetyl cysteine (2mM) under normoxia or hypoxia for 4 days. (C) Quantified crystal violet staining of shScramble H1975 cells after addition of glutathione under normoxia or hypoxia (n = 3). (D) Protein-normalize ATP levels in indicated shRNA cell line under normoxia or hypoxia (n = 9). (E) qPCR analysis of ETV4 mRNA levels in shACLY cells under normoxia or hypoxia (n = 6). (F) Western blot of ETV4 protein levels with ACLY knockdown under normoxia or hypoxia. (G) qPCR results of ETV1 and ETV5 mRNA levels in shACC1 cells under hypoxia or normoxia (n = 6). (H, I) GSEA analysis showing high overlap of genes changed with ETV4 and ACC1 (left panels) or ACLY (right panels) depletion. (H) Enrichment of ETV4-up-regulated genes in shACC1 (left panel) or shACLY (right panel) cells. (I) Depletion of ETV4-down-regulated genes in shACC1 (left panel) or shACLY (right panel) cells. Data are represented as mean values +/- SEM. All data are from the H1975 cell line.
(TIF)

S5 Fig ETV4 occupancy of the regulatory regions of PLEC and DUSP6. Modified UCSC Genome Browser and CistromeFinder interfaces showing ETV4 binding in the regulatory regions of (A) PLEC and (B) DUSP6. For both (A) and (B): (i) shows location of gene in genome; (ii) shows peaks of binding from ChIP-Seq data with ETV4 in PC3 cells, highlighted by red box; (iii) shows the annotated gene structures for each gene; (iv) shows abundance of acetylated-Histone H3 lysine 27 (H3K27Ac) at these locations; (v) dark bars to represent DNase hypersensitivity clusters at these genomic locations.
(TIF)

S6 Fig ACC1-altered genes likely represent both ETV4-dependent and -independent transcriptional targets. (A) Western blot showing overexpression of ETV4 in ACC1 depleted cells by 2 shRNAs. (B) qPCR analysis of a set of indicated genes whose ACC1-affected changes can be reversed with ETV4 expression, consistent with a pattern consistent of being downstream targets of ETV4 (n = 6). (C) qPCR analysis of a set of indicated genes whose changes could not be reversed with ETV4 expression, consistent with a pattern of not being downstream targets of ETV4 (n = 6). Data are represented as mean values +/- SEM. All data are from the H1975 cell line.
(TIF)

S7 Fig Metabolomics assay accurately reflects expected changes and the gene expression effects of different doses of α-KG. (A) Protein-normalized levels of palmitate measured in the indicated shRNA cell lines under normoxia (n = 3). (B, C) Protein-normalized levels of pyruvate (B) and lactate (C) in shScramble cells under normoxia and hypoxia (n = 3). (D) qPCR analysis of ETV4 mRNA levels after supplementation of α-KG at 3.5mM for 24 hours (n = 6). (E) Western blot of ETV4 protein levels after supplementation of α-KG at 3.5mM for 24 hours. (F, G) qPCR analysis of the changes of gene expression in the indicated genes after supplementation of different levels of α-KG for (F) up-regulated and (G) down-regulated genes (n = 6). (H) Western blot of HIF–1α protein levels after supplementation of α-KG at indicated doses.***** = p<0.0001. Data are represented as mean values +/- SEM. All data are from the H1975 cell line.
(TIF)

S8 Fig The effects of succinate and α-KG levels on the DNA methylation and epigenetic changes of cancer cells. (A) Quantified crystal violet stain of shACC1 and shScr cells supplemented with indicated dose of dimethyl-succinate under normoxia or hypoxia for 4 days (n = 3). (B) Quantified crystal violet stain of shACLY and shScr cells supplemented with indicated dose of dimethyl-succinate under normoxia or hypoxia for 4 days (n = 3). (C) qPCR analysis of the relative change in ETV4 mRNA levels in two shACC1 cells with the addition of DMSO or succinate (4mM). Ratio of 1 (dashed line) indicates no change with treatment (n = 6). (D) qPCR analysis of the relative change in ETV4 mRNA levels in two shACLY cells with the addition of DMSO or succinate (4mM). Ratio of 1 (dashed line) indicates no change with treatment (n = 6). (E) Percent of methylated CpG sites across the two shore regions and center of the ETV4 promoter CpG island as determined by bisulfite sequencing (n = 5). (F) Western blot analysis of indicated histone modification when supplemented with either DMSO or α-KG (1mM) for indicated length of time. (G) Western blot of indicated histone modifications with α-KG supplementation (3.5mM) for indicated length of time. Data are represented as mean values +/- SEM. All data are from the H1975 cell line.
(TIF)

S1 Table Multiple Hairpin Analysis of shRNA screens. Number of Multiple Hairpin Hit genes in genome-wide shRNA screens separated by number of short-hairpin RNAs (shRNAs) per gene.
(DOCX)

S2 Table Genes Passing Multiple Hairpin Analysis of shRNA screens. List of Multiple Hairpin Hit genes in genome-wide shRNA screens separated by effect of shRNA (“synthetic lethal” or “contextual survival”) and by stress (hypoxia or lactic acidosis)
(XLSX)

S3 Table RIGER Analysis of Hypoxia shRNA screen. Top 20 genes from RIGER analysis of log fold change and second best shRNA/gene in hypoxia “synthetic survival” category.
(DOCX)
