# Supplementary figures and images for: ACLY and ACC1 Regulate Hypoxia-Induced Apoptosis by Modulating ETV4 via α-ketoglutarate
Source: PLoS Genet. 2015 Oct 9;11(10):e1005599. doi: 10.1371/journal.pgen.1005599 (PMC4599891; doi:10.1371/journal.pgen.1005599)

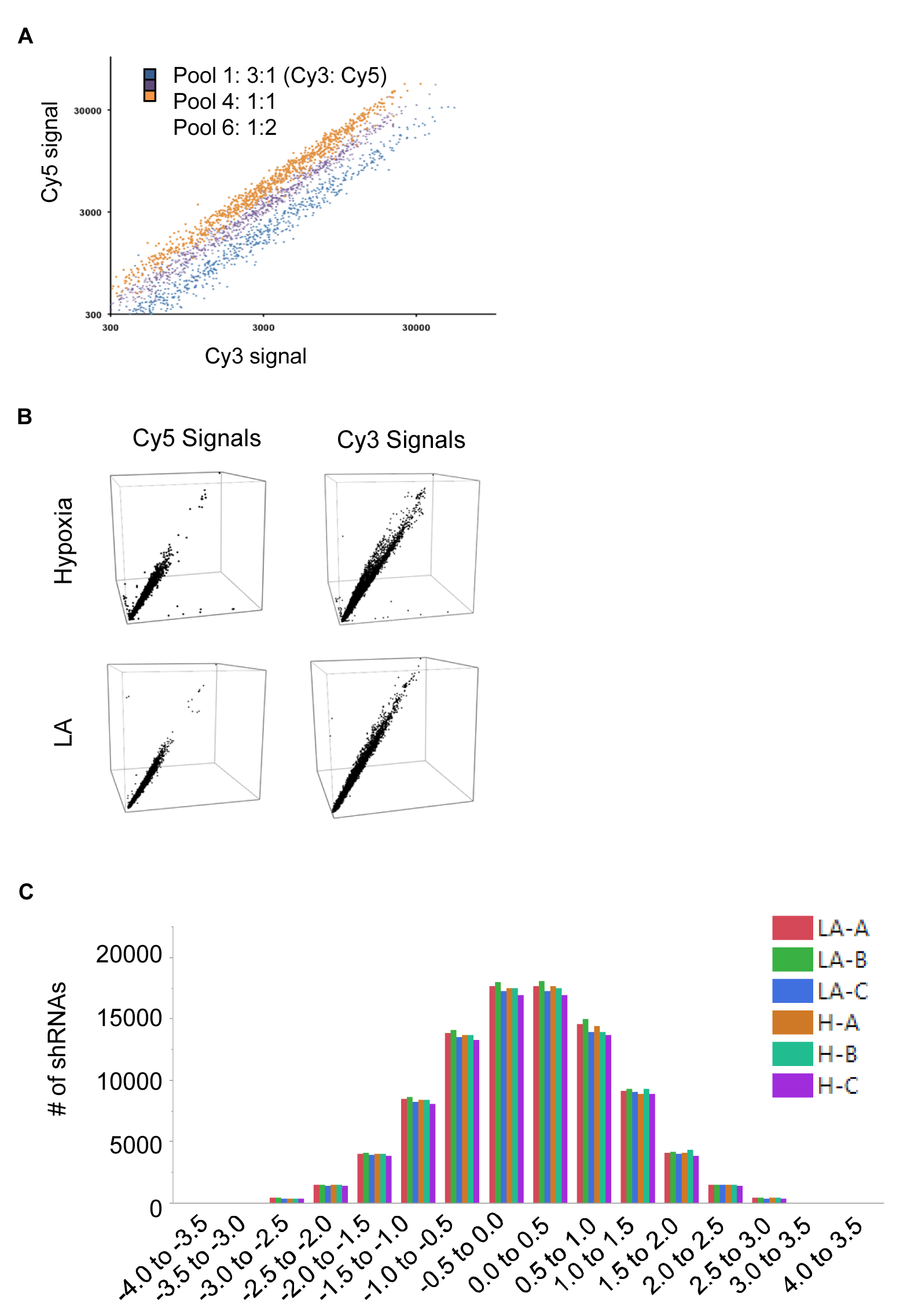

Supplement: S1 Fig — (A) Correlation plots showing PCR products, when mixed at different indicated ratios, are distinguishable by the custom microarray. (B) 3-D scatterplots showing reproducibility between biological triplicates’ of hypoxia and lactic acidosis treated samples for both Cy3 and Cy5 signals. (C) Distribution of R/G ratios by number of shRNAs, separated by treatment (LA = lactic acidosis, H = hypoxia) and replicate (n = 3). (TIF) [file pgen.1005599.s001.tif]

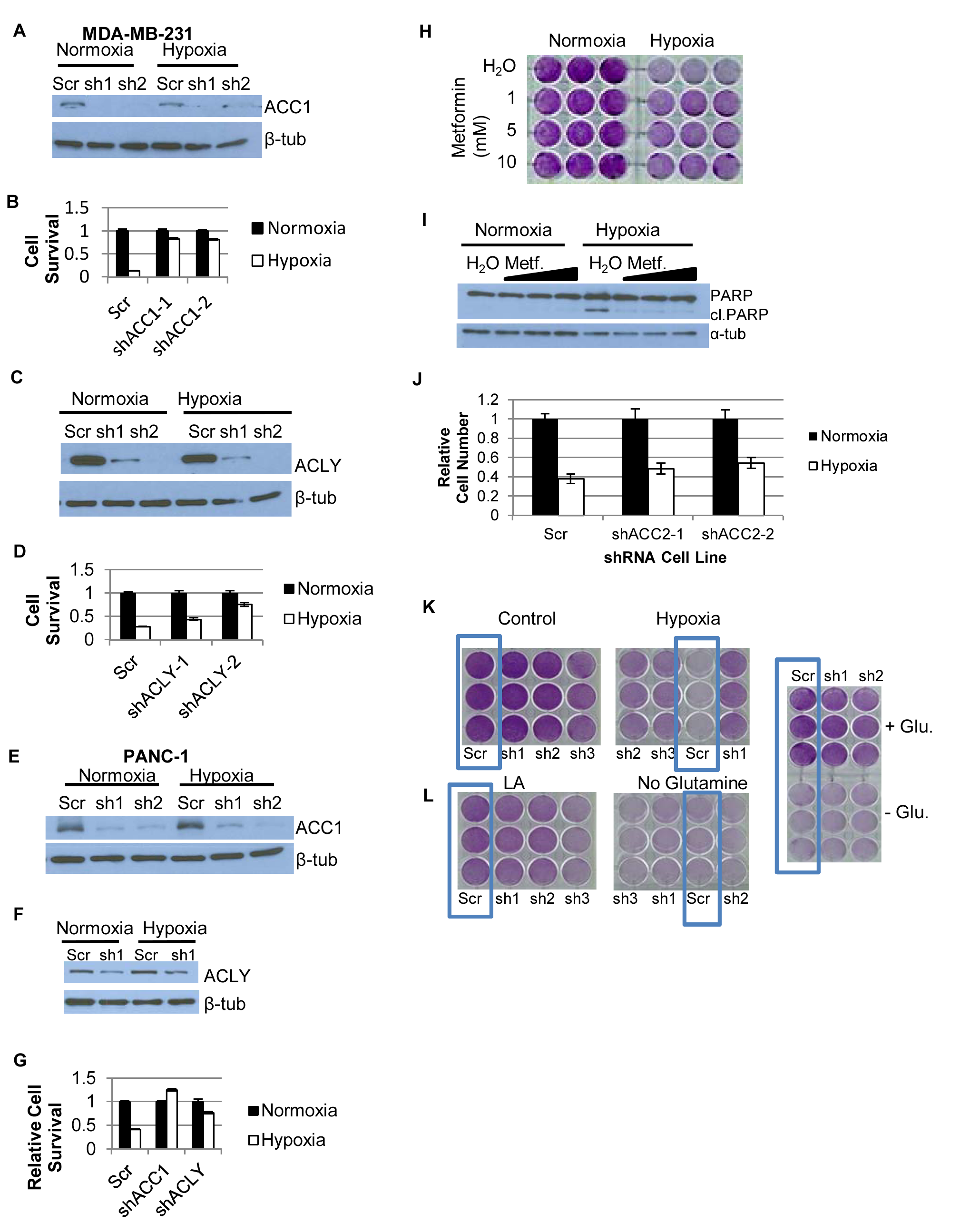

Supplement: S2 Fig — (A) Western blot of ACC1 protein knockdown by 2 shRNAs in MDA-MB–231 cells. (B) Quantified crystal violet of shACC1 MDA-MB–231 cells after 4 days of hypoxia (n = 3). (C) Western blot of ACLY protein knockdown by 2 shRNAs in MDA-MB–231 cells. (D) Quantified crystal violet of shACLY MDA-MB–231 cells after 4 days of hypoxia (n = 3). (E) Western blot of ACC1 protein knockdown in PANC–1 cells. (F) Western blot of ACLY protein knockdown in PANC–1 cells. (G) Quantified crystal violet staining of indicated shRNA PANC–1 cells after 6 days of hypoxia (n = 3). (H) Crystal violet staining of H1975 cells with simultaneous metformin treatment and hypoxia for 4 days. (I) Western blot of PARP in H1975 cells with hypoxia and metformin treatment. (J) Counts of viable cell number by trypan blue exclusion of shACC2 cells under normoxia or hypoxia for 4 days (n = 9). (K) and (L) Crystal violet of ACC1 and scramble (Scr) cells (boxed in blue rectangles) under indicated stresses (K-hypoxia, L- LA (lactic acidosis), no glutamine or no glucose (Glu)) for 4 days. Data are represented as mean values +/- SEM. (TIF) [file pgen.1005599.s002.tif]

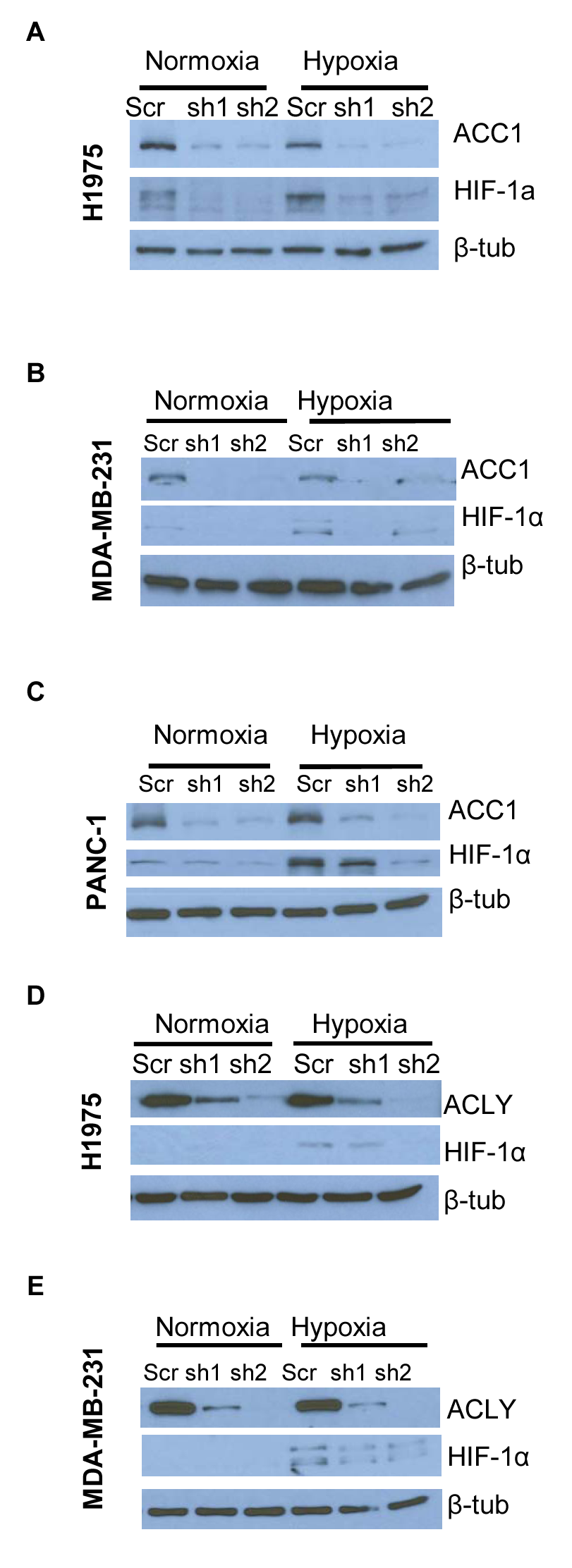

Supplement: S3 Fig — (A) Western blot of HIF–1α protein levels with ACC1 knockdown by 2 shRNAs in H1975 cells. (B) Western blot of HIF–1α protein levels with ACC1 knockdown by 2 shRNAs in MDA-MB–231 cells. (C) Western blot of HIF–1α protein levels with ACC1 knockdown by 2 shRNAs in PANC–1 cells. (D) Western blot of HIF–1α protein levels with ACLY knockdown by 2 shRNAs in H1975 cells. (E) Western blot of HIF–1α protein levels with ACLY knockdown by 2 shRNAs in MDA-MB–231 cells. (TIF) [file pgen.1005599.s003.tif]

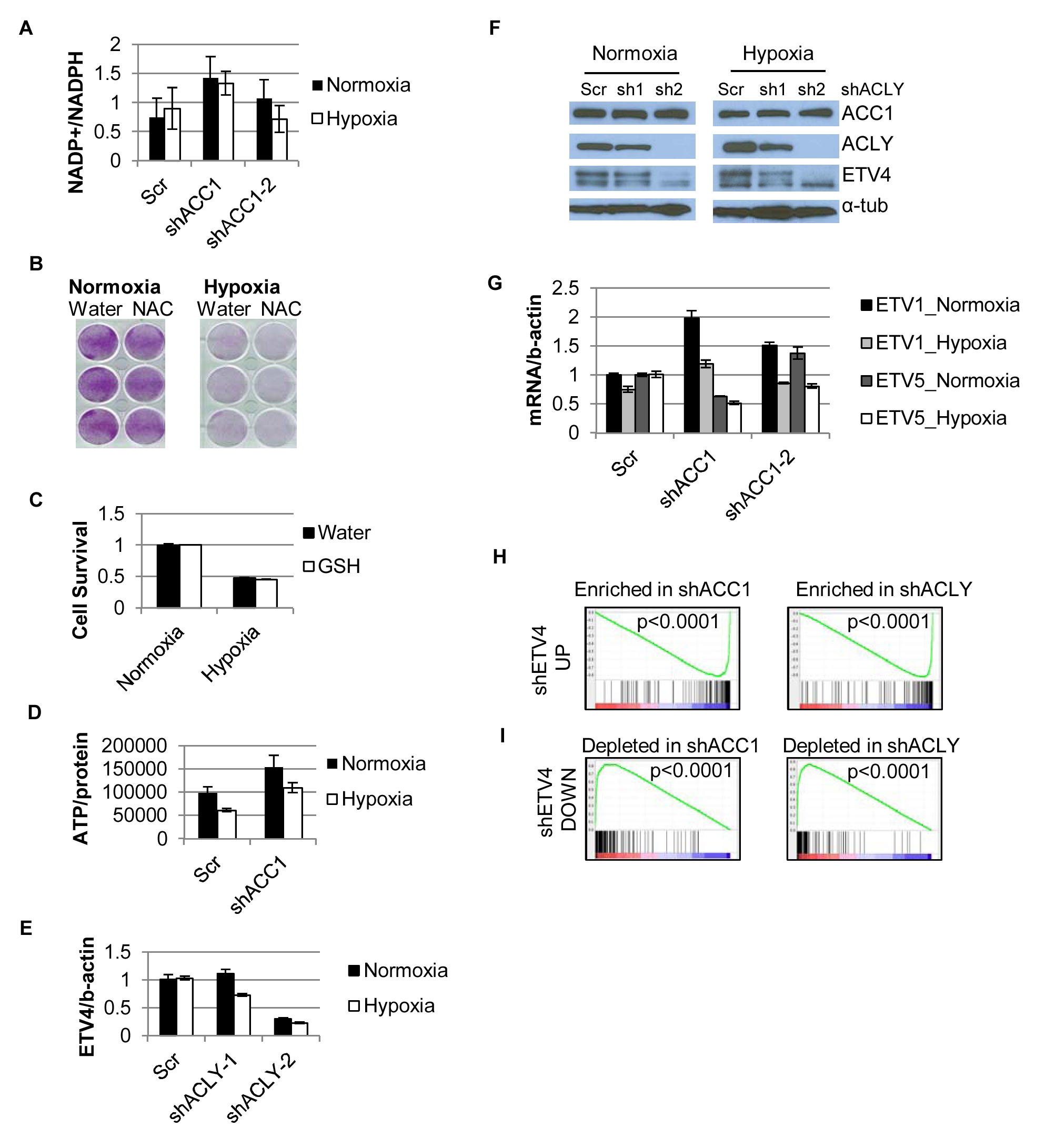

Supplement: S4 Fig — (A) NADP+/NADPH ratio under normoxia and hypoxia in shScr or shACC1 H1975 cells (n = 6). (B) Crystal violet staining of shScramble H1975 cells treated with N-acetyl cysteine (2mM) under normoxia or hypoxia for 4 days. (C) Quantified crystal violet staining of shScramble H1975 cells after addition of glutathione under normoxia or hypoxia (n = 3). (D) Protein-normalize ATP levels in indicated shRNA cell line under normoxia or hypoxia (n = 9). (E) qPCR analysis of ETV4 mRNA levels in shACLY cells under normoxia or hypoxia (n = 6). (F) Western blot of ETV4 protein levels with ACLY knockdown under normoxia or hypoxia. (G) qPCR results of ETV1 and ETV5 mRNA levels in shACC1 cells under hypoxia or normoxia (n = 6). (H, I) GSEA analysis showing high overlap of genes changed with ETV4 and ACC1 (left panels) or ACLY (right panels) depletion. (H) Enrichment of ETV4-up-regulated genes in shACC1 (left panel) or shACLY (right panel) cells. (I) Depletion of ETV4-down-regulated genes in shACC1 (left panel) or shACLY (right panel) cells. Data are represented as mean values +/- SEM. All data are from the H1975 cell line. (TIF) [file pgen.1005599.s004.tif]

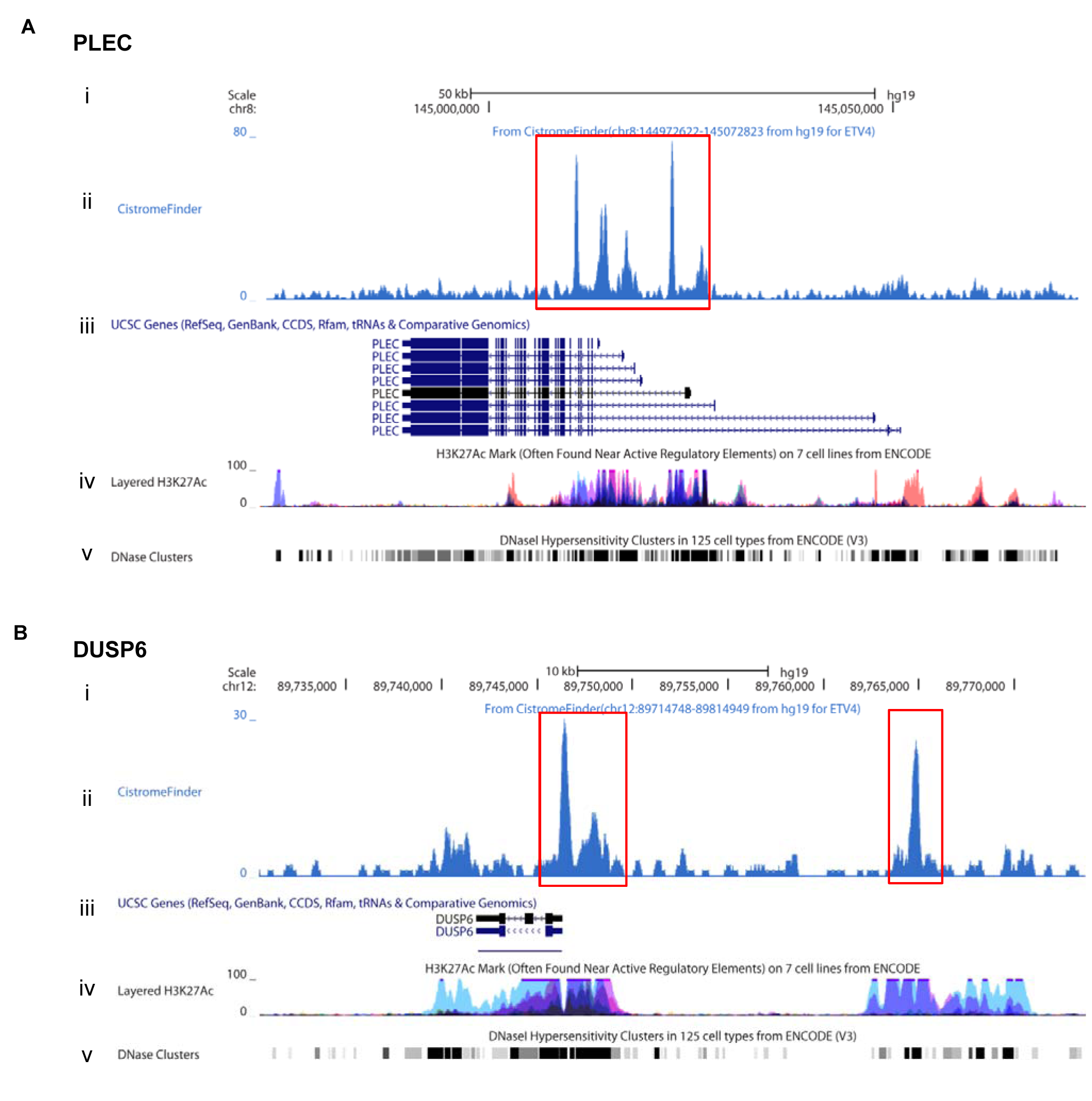

Supplement: S5 Fig — Modified UCSC Genome Browser and CistromeFinder interfaces showing ETV4 binding in the regulatory regions of (A) PLEC and (B) DUSP6. For both (A) and (B): (i) shows location of gene in genome; (ii) shows peaks of binding from ChIP-Seq data with ETV4 in PC3 cells, highlighted by red box; (iii) shows the annotated gene structures for each gene; (iv) shows abundance of acetylated-Histone H3 lysine 27 (H3K27Ac) at these locations; (v) dark bars to represent DNase hypersensitivity clusters at these genomic locations. (TIF) [file pgen.1005599.s005.tif]

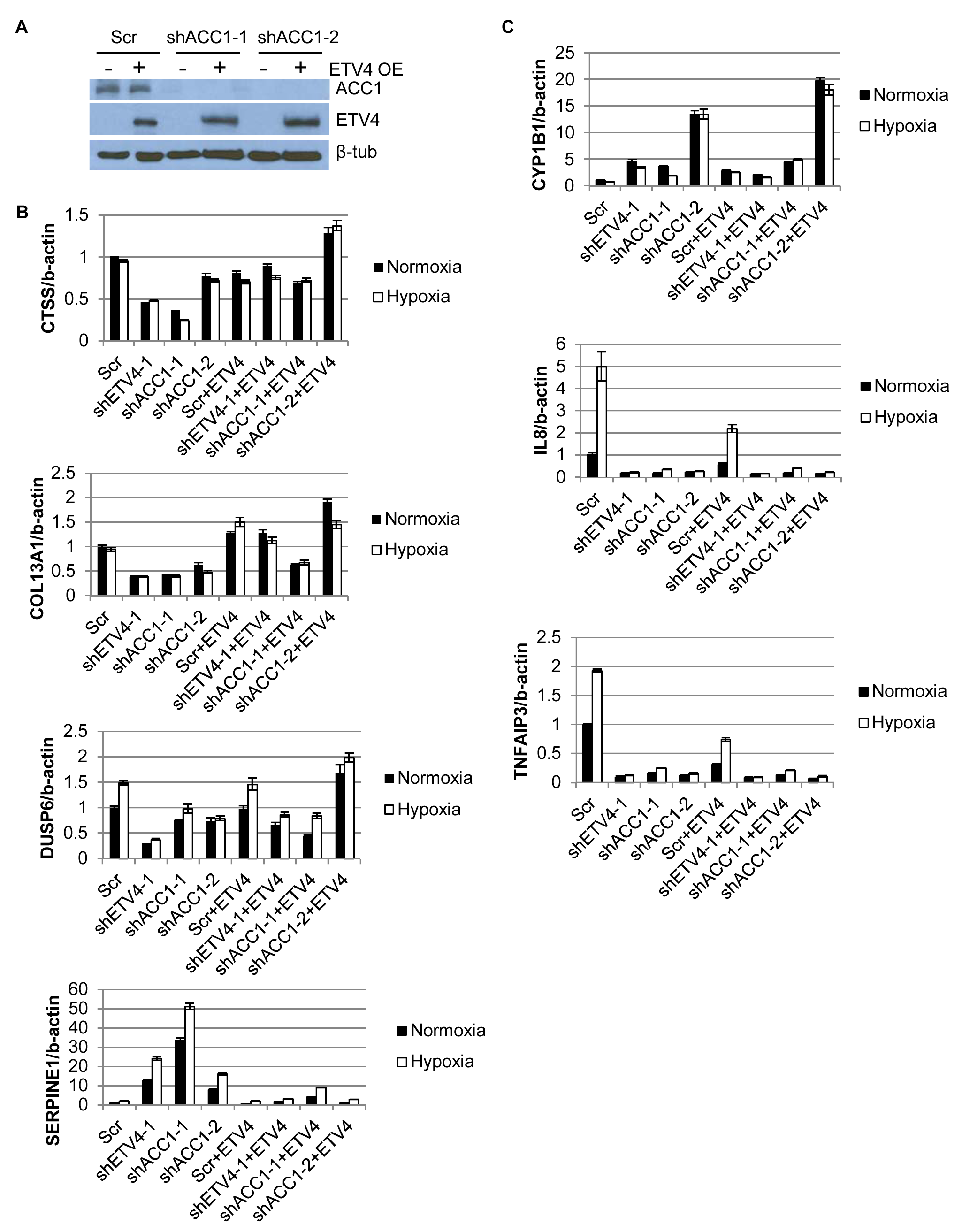

Supplement: S6 Fig — (A) Western blot showing overexpression of ETV4 in ACC1 depleted cells by 2 shRNAs. (B) qPCR analysis of a set of indicated genes whose ACC1-affected changes can be reversed with ETV4 expression, consistent with a pattern consistent of being downstream targets of ETV4 (n = 6). (C) qPCR analysis of a set of indicated genes whose changes could not be reversed with ETV4 expression, consistent with a pattern of not being downstream targets of ETV4 (n = 6). Data are represented as mean values +/- SEM. All data are from the H1975 cell line. (TIF) [file pgen.1005599.s006.tif]

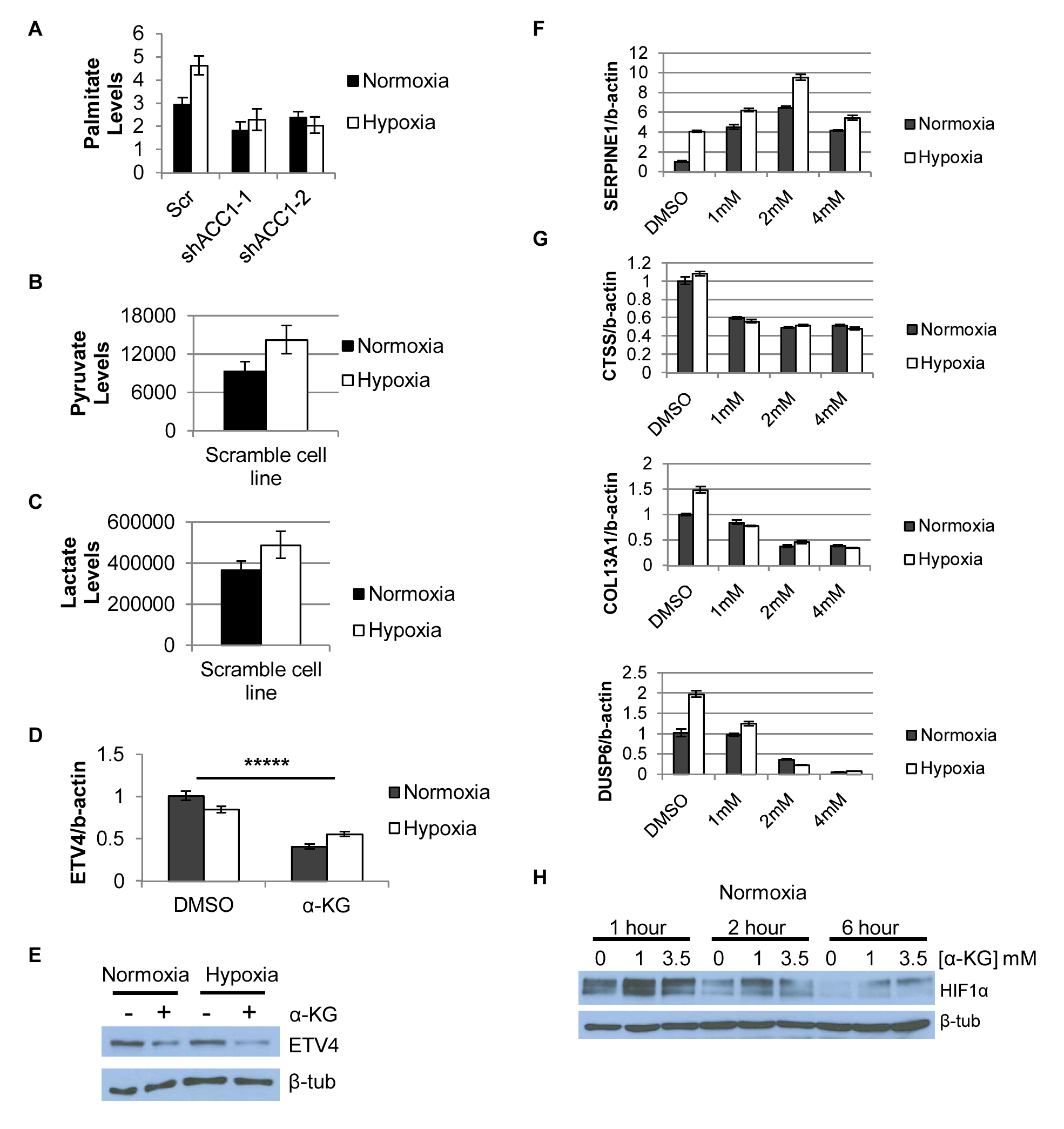

Supplement: S7 Fig — (A) Protein-normalized levels of palmitate measured in the indicated shRNA cell lines under normoxia (n = 3). (B, C) Protein-normalized levels of pyruvate (B) and lactate (C) in shScramble cells under normoxia and hypoxia (n = 3). (D) qPCR analysis of ETV4 mRNA levels after supplementation of α-KG at 3.5mM for 24 hours (n = 6). (E) Western blot of ETV4 protein levels after supplementation of α-KG at 3.5mM for 24 hours. (F, G) qPCR analysis of the changes of gene expression in the indicated genes after supplementation of different levels of α-KG for (F) up-regulated and (G) down-regulated genes (n = 6). (H) Western blot of HIF–1α protein levels after supplementation of α-KG at indicated doses.***** = p<0.0001. Data are represented as mean values +/- SEM. All data are from the H1975 cell line. (TIF) [file pgen.1005599.s007.tif]

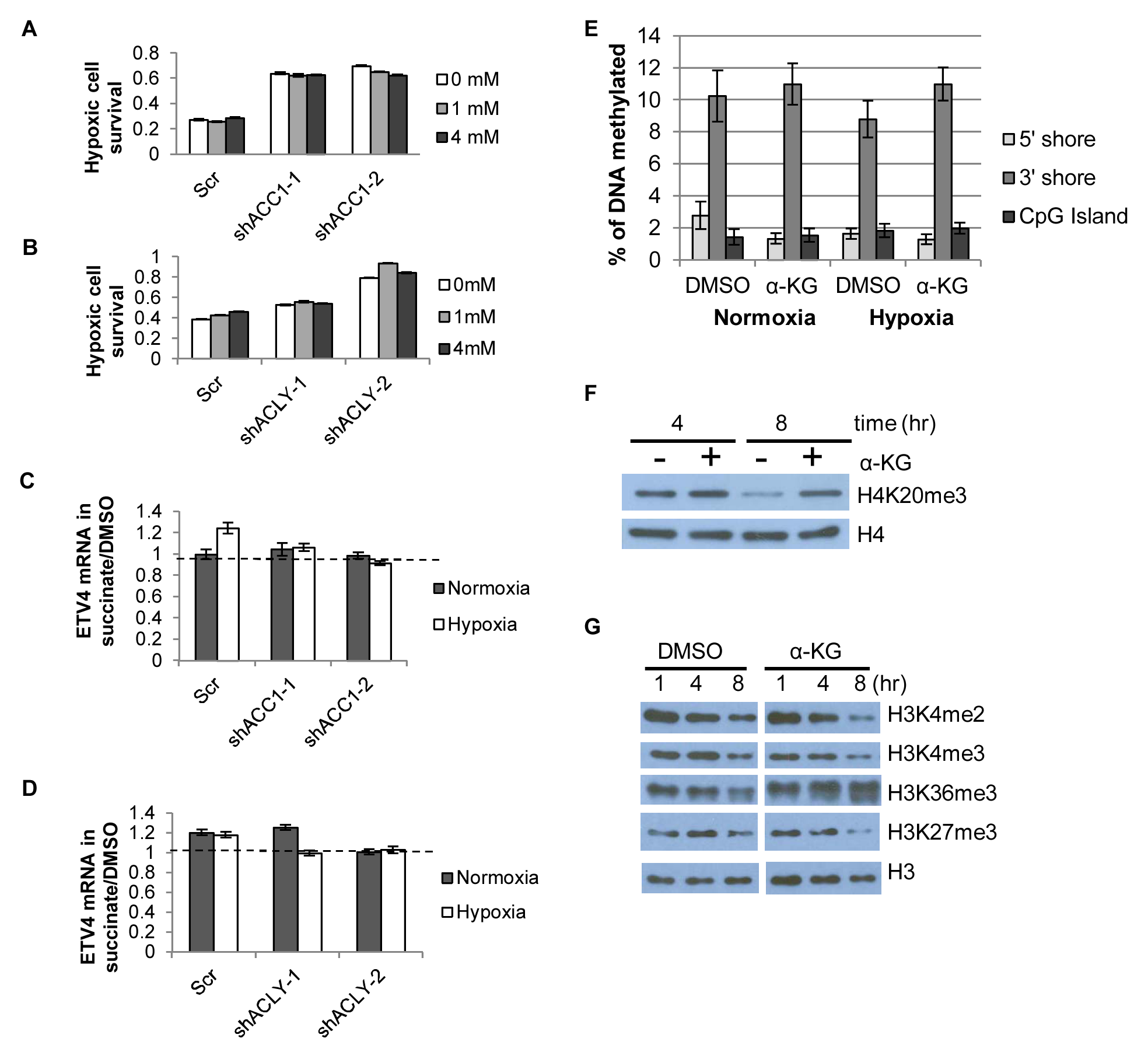

Supplement: S8 Fig — (A) Quantified crystal violet stain of shACC1 and shScr cells supplemented with indicated dose of dimethyl-succinate under normoxia or hypoxia for 4 days (n = 3). (B) Quantified crystal violet stain of shACLY and shScr cells supplemented with indicated dose of dimethyl-succinate under normoxia or hypoxia for 4 days (n = 3). (C) qPCR analysis of the relative change in ETV4 mRNA levels in two shACC1 cells with the addition of DMSO or succinate (4mM). Ratio of 1 (dashed line) indicates no change with treatment (n = 6). (D) qPCR analysis of the relative change in ETV4 mRNA levels in two shACLY cells with the addition of DMSO or succinate (4mM). Ratio of 1 (dashed line) indicates no change with treatment (n = 6). (E) Percent of methylated CpG sites across the two shore regions and center of the ETV4 promoter CpG island as determined by bisulfite sequencing (n = 5). (F) Western blot analysis of indicated histone modification when supplemented with either DMSO or α-KG (1mM) for indicated length of time. (G) Western blot of indicated histone modifications with α-KG supplementation (3.5mM) for indicated length of time. Data are represented as mean values +/- SEM. All data are from the H1975 cell line. (TIF) [file pgen.1005599.s008.tif]
