# Supplementary material for: ACLY and ACC1 Regulate Hypoxia-Induced Apoptosis by Modulating ETV4 via α-ketoglutarate
Source: PLoS Genet. 2015 Oct 9;11(10):e1005599. doi: 10.1371/journal.pgen.1005599 (PMC4599891; doi:10.1371/journal.pgen.1005599)
Supplement: S1 Table — Number of Multiple Hairpin Hit genes in genome-wide shRNA screens separated by number of short-hairpin RNAs (shRNAs) per gene. (DOCX) [file pgen.1005599.s009.docx]

**S1 Table. Multiple Hairpin Analysis of shRNA screens.** Number of Multiple Hairpin Hit genes in genome-wide shRNA screen separated by number of short-hairpin RNAs (shRNAs) per gene.

| Category of shRNA | Number of Hairpins | Lactic Acidosis | Hypoxia |
| --- | --- | --- | --- |
| shRNAs Enriched in stress | | | |
|  | 2 | 83 | 158 |
|  | 3 | 3 | 13 |
|  | 4 | 0 | 2 |
|  | total | 86 | 173 |
| shRNAs Depleted in stress | | | |
|  | 2 | 83 | 114 |
|  | 3 | 3 | 7 |
|  | 4 | 0 | 1 |
|  | total | 86 | 122 |
