# Supplementary material for: ACLY and ACC1 Regulate Hypoxia-Induced Apoptosis by Modulating ETV4 via α-ketoglutarate
Source: PLoS Genet. 2015 Oct 9;11(10):e1005599. doi: 10.1371/journal.pgen.1005599 (PMC4599891; doi:10.1371/journal.pgen.1005599)
Supplement: S3 Table — Top 20 genes from RIGER analysis of log fold change and second best shRNA/gene in hypoxia “synthetic survival” category. (DOCX) [file pgen.1005599.s011.docx]

**S3 Table . RIGER Analysis of Hypoxia shRNA screen.** Top 20 genes from RIGER analysis of log fold change and second best shRNA/gene in hypoxia “synthetic survival” category.

| RANK | Gene Symbol | second best shRNA sequence | 1/NES |
| --- | --- | --- | --- |
| 1 | HSPE1 | TTAGAACCCGATCCAACAGCGA | 449.8426 |
| 2 | NUDT21 | AATATGTGCAGGAATATATGGATGC | 138.6001 |
| 3 | UBE1 | TATAGCCACTGCATGATGGGCA | 108.1666 |
| 4 | LANCL3 | TTTAGAGTTTCCCGTGAGCCGG | 102.3856 |
| 5 | C11orf71 | TTGGGTAAGGTGAGAATCTGGC | 101.6363 |
| 6 | NCKAP1L | TAAGCTTGTCAGTCTCCTGGCC | 67.11409 |
| 7 | DNAJC6 | TATAGCAATCGAATGGCTGGGC | 56.56109 |
| 8 | PLK1 | TATAGCCAGAAGTAAAGAACTCTGC | 56.33803 |
| 9 | UBA52 | TTTGACCTTCTTCTTGGGACGC | 55.67929 |
| 10 | STT3B | TAATGTCTCCCTGTTATCAGGT | 50.65856 |
| 11 | RPL23 | TTTATTGTTCACTATGACTCCTTGC | 49.48046 |
| 12 | NRCAM | TTAACTGGATATTTACCACCCTTGC | 45.89261 |
| **13** | **ACACA** | **AATAATCTTAAGGTCATGTGGATGC** | **41.98153** |
| 14 | ARCN1 | TACAGTTTCTCCATAGGCTGGT | 40.63389 |
| 15 | AP1S1 | TTGTCTTGGCCCTCGATGGCGC | 38.58025 |
| 16 | XRN1 | TATGATGTTGATTTACTTGGCTTGC | 38.05175 |
| 17 | C15orf2 | TTATACTCAGGGCTCTTCTCCA | 37.67898 |
| 18 | NUP210 | TTACCAAGGCAACAGAGGAGGG | 37.59398 |
| 19 | NUP43 | ATTTACAGTAAGAATCTCAGGATGC | 35.89375 |
| 20 | NOTCH3 | ATACAGATACAGGTGAACTGGC | 35.77818 |
